# DataXflowGen for GenAI-driven model generation

**DOI:** 10.1038/s41598-026-56492-8

**Published:** 2026-06-06

**Authors:** Samantha A. W. Crouch, Tim Breitenbach

**Affiliations:** Department of Bioinformatics, Biocenter, University of Würzburg, Am Hubland, 97074 Würzburg, Germany

**Keywords:** Signed GRNs, DataXflow, GenAI, LLMs, Computational biology and bioinformatics, Mathematics and computing, Systems biology

## Abstract

For years, mathematical models have been successfully used to explain biological, chemical, or physical relationships. The enormous advances in artificial intelligence in understanding context-specific content can support humans in generating interaction hypotheses for modeling, enabling models to be generated and modified far more quickly. Our pipeline, DataXflowGen, uses GenAI to create models based on information such as research publications available on the internet. This approach reduces the need for specialized knowledge, as GenAI identifies relevant research papers online and extracts the relevant information. GenAI can build a model that fits the data to test its hypothesis. As a result, humans only need to conduct an in-depth analysis of those model components that do not align with the data, saving time when building a suitable regulatory network based on existing knowledge. In summary, GenAI is used to generate a human-interpretable model as a hypothesis, allowing an understanding of therapy suggestions and an explanation of actions based on a model validated with specific data, avoiding black-box AI decisions. This constitutes an approach to explainable AI that supports humans in analyzing complex relationships.

**Supplementary Information:**

The online version contains supplementary material available at 10.1038/s41598-026-56492-8.

## Introduction

Modern science and technology today rely heavily on artificial intelligence (AI), which is becoming increasingly influential, especially in the natural sciences. The relevance of AI is demonstrated by the fact that there are already over 342,883 (12/19/2025) specialist articles on PubMed with the term “artificial intelligence” in the title^1^. The application of AI for skin cancer diagnosis, as described by Chanda et al.^2^, or the utilization of AlphaFold 3 for predicting protein structures via GenAI^3^, exemplifies remarkable advancements. Predictive AI, machine learning (ML), deep learning, explainable AI (XAI), and generative AI (GenAI) are only a few of the subcategories that comprise AI, which is no longer a single, unified concept^3–6^. The present use of GenAI, represented by AI assistants like Perplexity and ChatGPT based on Large Language Models (LLMs) or even on multimodal models like vision large language models (VLM)^7^, has become established in society. The continuous advancements of these AI assistants can represent a major step forward in the medical field^8,9^. Prompt engineering allows LLMs with internet access to perform rapid searches based on specific, predefined criteria. Numerous publications attempt to explain these “precise” instructions so that the prompt query delivers an accurate result^3,10–13^. The wide range of applications currently available, from data analysis and the evaluation of medical image data to automatic hypothesis formation, is reflected in the diversity^2,14,15^. An interesting study comparing the diagnostic abilities of GenAI with those of doctors found that GenAI was not better than doctors^16^. GenAI showed promising diagnostic capabilities, but its performance depended on the accuracy of the models used^16^.

Mathematical modeling constitutes the essential framework of systems biology by representing intricate biological processes, ranging from gene regulation to signaling pathways to metabolic networks, through formal equations and graph theory^17,18^. With the introduction of AI, this technology is undergoing a revolutionary expansion: GenAI models are now capable of evaluating omics datasets, uncovering hidden interaction patterns, and independently building entire interaction networks. Tools, such as GRNIX, generate gene regulatory networks (GRNs), and BIND accurately predicts molecular connections. This demonstrates how AI not only replicates existing networks but also creates new biological connections, however, without taking activating or inhibiting interactions into account^19,20^. Two other methods, GRNPT and LLM4GRN, use large language models to construct GRNs. GRNPT uses LLM-based text embeddings as a prior, while LLM4GRN directly evaluates gene pairs for regulatory edges using a language model^21,22^. Both methods only provide answers as to whether regulation is present. However, DataXflowGen goes one step further by using GenAI to indicate the direction of regulation for each gene–gene interaction as activation or inhibition. This information is important for our pipeline because it allows us to fit a model to test the AI hypothesis and thus protect us from hallucinations. Subsequently, such a validated model can be used to make predictions about, e.g., therapy and calculate optimal interventions with our external stimuli framework (see also^23^) in an explainable manner.

While challenges such as interpretability and data integration continue to require interdisciplinary innovation, customized medicine and synthetic biology are being driven by this combination of mathematical accuracy and AI-powered creativity^19,24,25^.

While the advantageous attributes primarily resulting from using AI are obvious, the associated issues cannot be avoided^26^. An article published in Nature Medicine by Thirunavukarasu describes how the biggest limitations of large language models can result from their limited timeliness, potential errors in accuracy, inconsistencies between responses, ethical concerns (e.g., bias and misinformation), lack of transparency of the models, and their limited interpretability^27^. A systematic review by Shool et al. explains the importance of properly integrating AI assistants into clinical workflows to assess reliability, safety, and ethical alignment in clinical medicine^9^. Narayanan and Kapoor highlight the issues arising from the lack of critical examination of AI and the absence of error logging throughout its implementation^28^. Cybersecurity is another critical factor that must not be disregarded while utilizing GenAI in the healthcare sector^26^. Critical questioning of AI will be the new challenge of our time^27^.

Since the use of GenAI could accelerate and simplify the generation of signed gene regulatory networks (sgGRNs), thereby enabling a fundamental understanding of complex functions, we introduce “DataXflowGen”, an extension of “DataXflow”^23^. This extension uses LLM-based GenAI (PerplexityAI^29^) to create sgGRNs examplified with single-cell data that changes over time from senescence experiments. Our work innovates by combining GenAI with existing software (DataXflow^23^) to automate the generation of hypotheses about gene interactions in specific contexts. This approach validates hypothesized GRNs and identifies areas where GenAI fails in suggesting data-consistent gene interactions. Thus, this combination helps to review GenAI output and creates a feedback system that leads to a more effective GRN generation.

DataXflowGen is user-friendly and can also be used by those without extensive programming knowledge. Given the limitations, such as hallucinations of generative artificial intelligence^26^, Data2Dynamics (D2D)^23,30^ is helpful in our pipeline to facilitate the verification of the generated interaction hypothesis based on available data. Our new approach aims to automate the validation of generated hypotheses based on measured data, thereby paving the way for agent-based model development by establishing a feedback loop based on the model’s deviation from the data. Based on gene interaction networks, therapeutic predictions can be made. DataXflowGen allows faster model generation that is not limited to use in cancer or aging research.

We remark that this work is not limited to protein or RNA interaction networks. GRNs can be used generally to model any kind of interaction between genes, such as methylation. It might require several edges between the same node, or that there are several nodes representing the corresponding RNA, protein, or methylation activity. The techniques presented in this work include such concepts.

## Results

In this section, we integrated generative AI (GenAI) with data-driven modeling to iteratively create a signed Gene Regulatory Network (sgGRN) for senescence based on the Wechter et al. data^31^. In order to achieve this, we added GenAI (Perplexity) steps into our DataXflow pipeline^23^ to produce models faster and more autonomously. The following results visualize two different ways to get the best model.

### Iterative modeling via GenAI

**Fig. 1:**
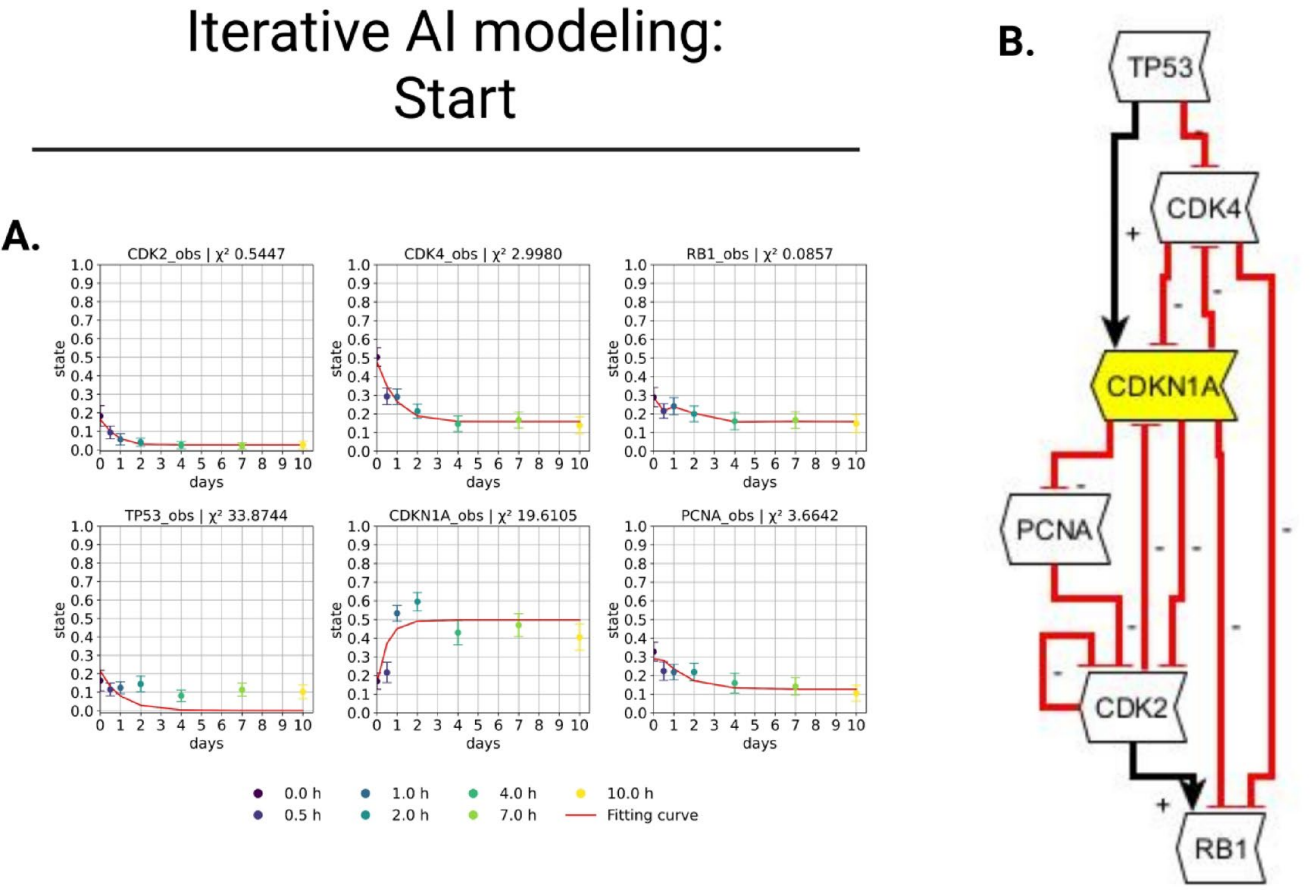
Senescence Start-Model. (**A**) Result of first fitting. Ten time points (days) are available for all 6 genes with their representative state (between 0 and 1): 0 d (dark purple), 0.5 d (purple), 1 d (blue), 2 d (turquoise), 4 d (green), 7 d (light green), 10 d (yellow). We provided Chi-2 values for each gene to evaluate the precision of the fit for the expression data (red line). Total Chi-2 value equals 60.77 with 42 data points and 28 free parameters. (**B**) First topology with 6 regulatory nodes. Positive regulation is represented by black arrows, whereas negative regulation is indicated by red inhibitory arrows. The node outlined in yellow represents positive regulation by ethylene oxide (ETO) treatment for the model. All white nodes and regulations are based on GenAI responses. Created in BioRender. Crouch, S. (2026) https://BioRender.com/tw9l06o.

Iterative modeling is a method for iteratively incorporating genes (nodes) into a model based on respective chi-square (Chi-2) values that are a measure of the goodness-of-fit of the existing genes in the model with respect to experimental data. The senescence marker Cyclin Dependent Kinase Inhibitor 1A (CDKN1A), linked to senescence and regulated by Ethylene Oxide (ETO) treatment^31^, served as an initial node to develop this senescence model (Fig. 1; yellow node).

Based on the GenAI request (first prompt, Fig. S6 in the supplement), the five genes identified as involved in fibroblast senescence and interacting with CDKN1A include Cyclin Dependent Kinase 2 (CDK2), Cyclin Dependent Kinase 4 (CDK4), RB Transcriptional Corepressor 1 (RB1), Tumor Protein P53 (TP53), and Proliferating Cell Nuclear Antigen (PCNA) (Fig. 1B). According to GenAI’s response (second prompt, Fig. S8, supplement), the interactions (inhibiting (− ; red) and activating (+ ; black)) were defined. The model’s fit to the data was then evaluated using parameter fitting (D2D step in our DataXflow pipeline^23^), and the outcomes are presented in Fig. 1A. Seven distinct measurement times, ranging from 0 to 10 days (0 d (dark purple), 0.5 d (purple), 1 d (blue), 2 d (turquoise), 4 d (green), 7 d (light green), and 10 d (yellow)), are assigned to each gene with their representative state (between 0 and 1 where 1 represents the maximum expression value of the corresponding gene in the dataset). The graphs and their Chi-2 values, which are displayed above the plots, illustrate how well the data correspond to the model. With 42 data points and 28 free parameters, the first experiment revealed that the GenAI-generated model had a total Chi-2 value of 60.77.

**Fig. 2:**
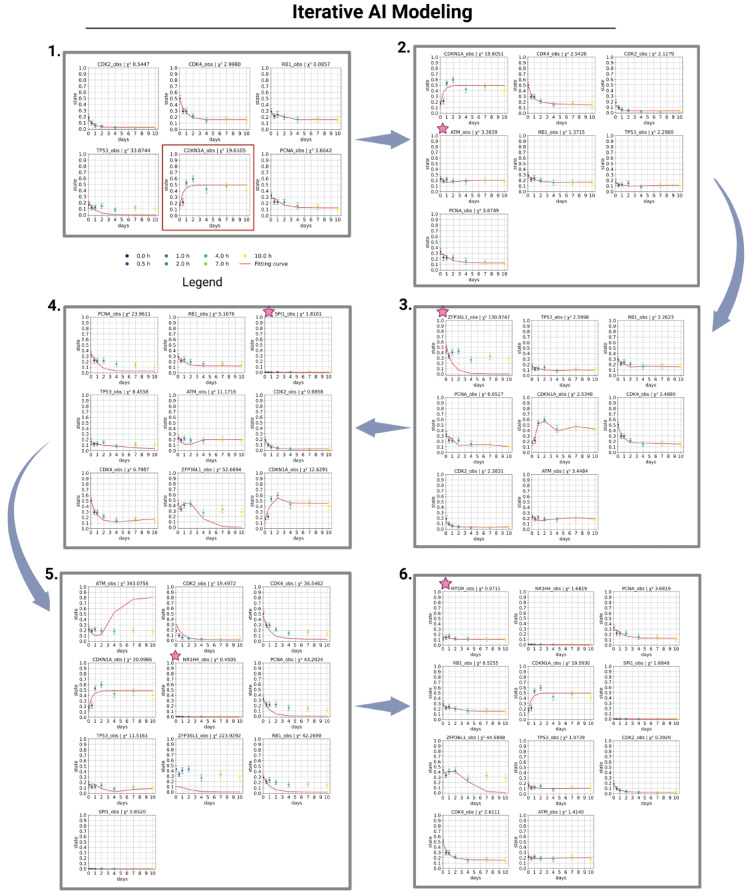
Iterative GenAI Modeling. Result of six iterative fitting steps. Ten time points (days) are available for all genes for each plot with their representative state (between 0 and 1): 0 d (dark purple), 0.5 d (purple), 1 d (blue), 2 d (turquoise), 4 d (green), 7 d (light green), 10 d (yellow). We provided Chi-2 values for each gene to evaluate the precision of the fit for the expression data. The pink stars signify newly incorporated genes (nodes) according to the GenAI conclusion. In the initial plot (1.), all genes were incorporated based on the GenAI response, except CDKN1A (shown by the red box, human-based information^31^) (refer to Fig. 1). All plots are presented individually, accompanied by their topology and total Chi-2 values in the appendix. Created in BioRender. Crouch, S. (2026) https://BioRender.com/h2b3xp4.

Chi-2 is specified for each gene and serves as an indication of how well the model fits the data. A big Chi-2 value can indicate the necessity to identify an additional regulatory influence to regulate this gene (node). The gene in the network requiring additional activation or inhibition can be identified via the individual Chi-2 values. Figure 2 depicts an iterative procedure for developing a larger model. According to the Chi-2 data, the GenAI feedback loop (prompt in the supplement, Fig. S9) identifies novel interacting genes and defines every possible interaction within the network for the new gene (node). With a Chi-2 value of 33.87, the gene of concern is *TP53* (Fig. 2). Based on this data, GenAI contributed to further optimizing the model fitting through a back-loop. The results of each iterative step can be found in the supplement (Figs. S1 to S5).

**Fig. 3:**
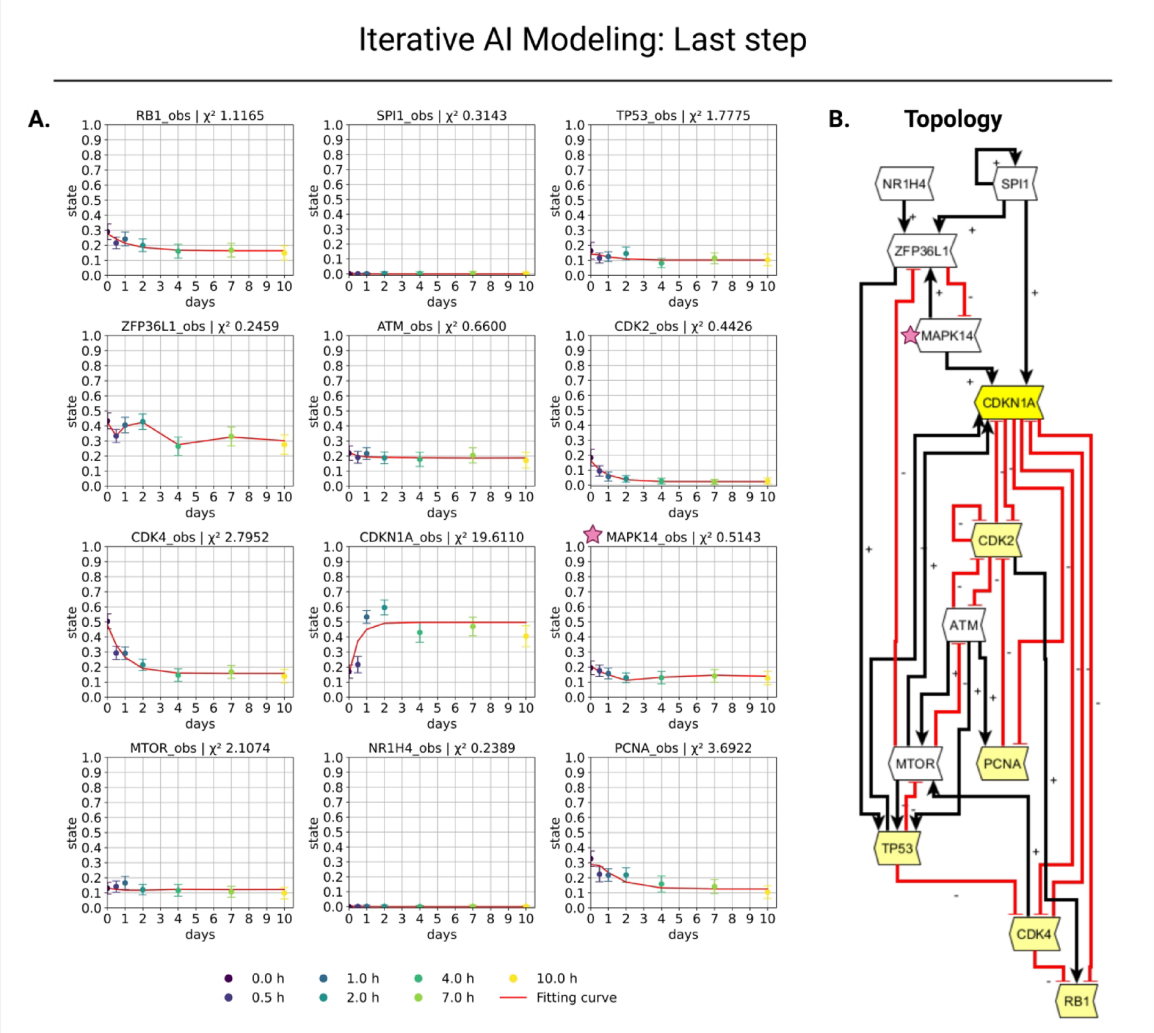
Final result. (**A**) Twelve regulatory nodes define the topology of an interaction model (Red inhibitory arrows indicate negative regulation, and black arrows show positive regulation). Positive regulation by the ethylene oxide (ETO) treatment of the model is represented by the nodes highlighted in green (for further details, see “Methods” section). (**B**) The 12-node adaptation’s outcomes are displayed. Each gene has 10 time points (in days) with corresponding expression values ranging from 0 to 1. Dark violet (0 d), violet (0.5 d), blue (1 d), turquoise (2 d), green (4 d), light green (7 d), and yellow (10 d) are the colors on the color scale. (**B**) To evaluate how well the expression data fit the model, Chi-2 values were computed for every gene. With 84 data points and 55 free parameters, the total Chi-2 value is 33.51 (*p* = 0.257) compared to the total Chi-2 value in the first step, which was 60.77 with 42 data points and 28 free parameters. The model has improved based on the Chi-2 value. Created in BioRender. Crouch, S. (2026) https://BioRender.com/9pn5i4z.

The final step of iterative modeling presents the last outcome of the model development (Fig. 3). Six nodes (ATM, ZFP36L1, SPI1, NR1H4, MTOR, and MAPK14; added in the order specified, marked with pink asterisks in the figures) were systematically incorporated into the senescence model. The final model (Fig. 3) demonstrates a great fit to the data with a total Chi-2 of 33.51 and a *p*-value *p* = 0.257. The global fit is therefore unremarkable at the 5% level of significance (Chi-2/dof = 1.16). To classify the model complexity and as a pure usability check for the following analyses, a L1 (Lasso) regularization and reduction analysis was then performed for evaluation (see supplement: L1 regularization and re-fitting).

27 parameters with values below the predefined threshold (|*p*|< 10) were fixed, and a refit was performed on the reduced structure without L1 regularization (nParam = 28; dof = 56). In this step, the most important parameters for the model with L1 regularization were determined. The fit remained virtually unchanged (before refit: Chi-2 = 33.51, after refit: Chi-2 = 33.28), while all interactions considered were retained (4/4; kept_interaction.csv is provided at: https://github.com/Sunbio1/DataXflowGen). This suggests that the central interactions are robust and data-driven, and that parameter adjustments are not modeled by a large number of meaningless interactions.

How well the data fit the model is displayed in Table SR1 in the supplement. The table summarizes the total Chi2 value, data points, free parameters, model fit, and added genes for each fit based on the gene with the highest Chi-2 value. Our model in step 2 also fits the data well (Chi-2 = 34.91) (Fig. 2, Table SR1). Both models can be suitable for future investigations, such as for the external stimuli framework. In the present study, we aimed to demonstrate the model generation; therefore, we extended the initial model to achieve the next optimal fit. This outcome demonstrates the value of GenAI in creating novel models derived from data.

### One-shot modeling for generating models based only on genes of interest

One-shot modeling is a method developed to rapidly produce a model using an extensive list of genes of interest. This approach represents another variant of sgGRN generation. The GenAI gene names are provided in a list determined to be significant for the model or derived from preliminary analyses, such as a differentially expressed genes (DEG) list or machine learning findings^32–35^. Optionally, the GenAI subsequently augments this list with a predetermined number of genes that interact with the specified genes and align with the requested background. This is often necessary because genes from a DEG analysis, for example, do not automatically interact. In this case, a previous pathway analysis could be helpful. While it does not identify interaction partners, it reveals pathway specificities. In this case, the first prompt (Fig. S6, sup) of the initial modeling pipeline can be extended to a set of genes or even just to specific signaling pathways.

**Fig. 4:**
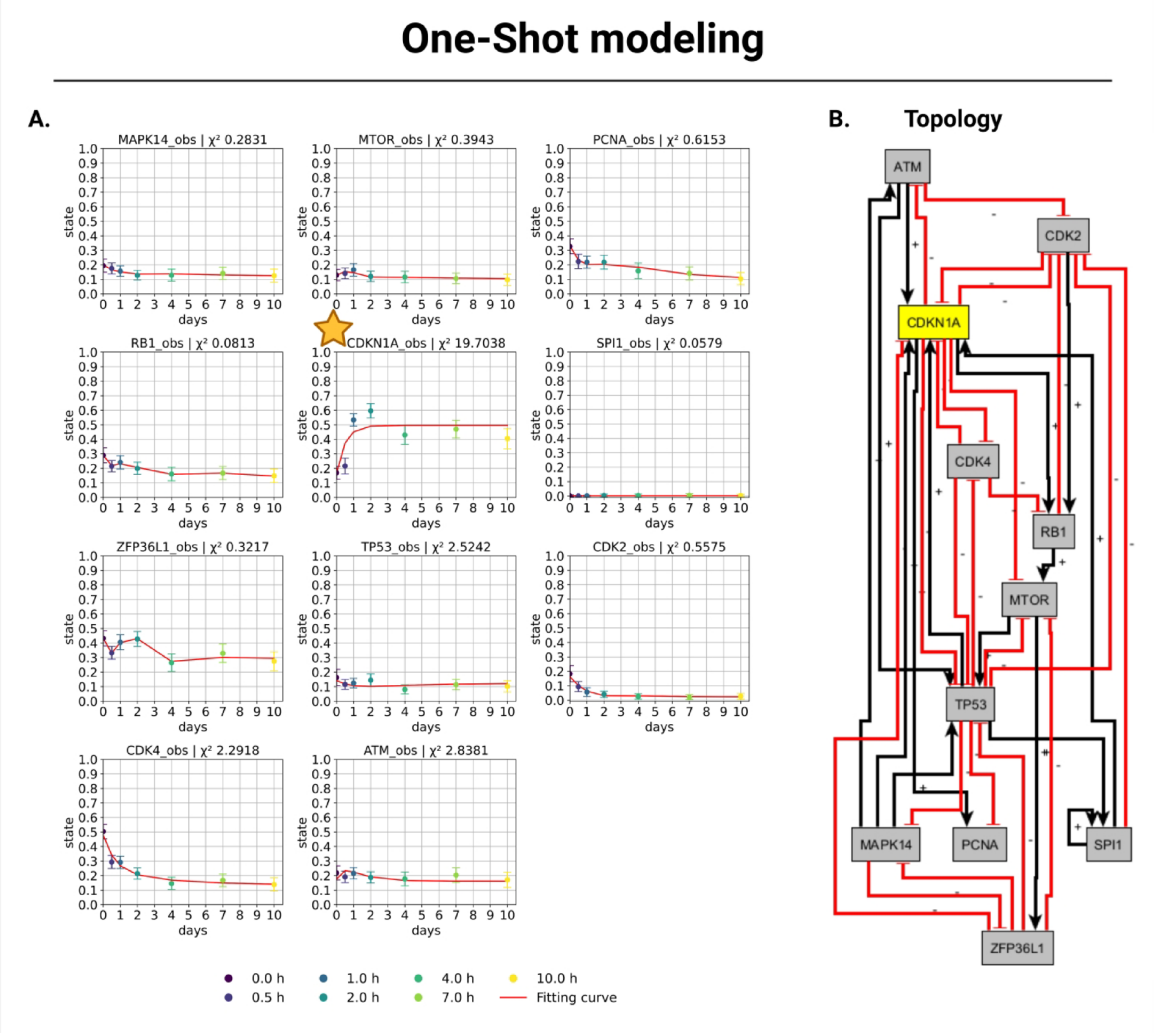
One-shot Senescence Model. (**A**) Results of model fitting. Ten time points (days) are available for each gene with their representative state (between 0 and 1): 0 d (dark purple), 0.5 d (purple), 1 d (blue), 2 d (turquoise), 4 d (green), 7 d (light green), 10 d (yellow). We provided Chi-2 values for each gene to evaluate the precision of the fit for the expression data. An orange star marks the gene that is of interest for the next step in iterative modeling. Overall, the model fits well with Chi-2 = 29.16 (77 data points, 32 free parameters), with *p* = 0.9676 (dof = 45) and *p* = 1.0000 (dof = 77) consistent with the data at the 95% level after L1 regularization. (**B**) Topology of the interaction model with 12 regulatory nodes. Positive regulation is shown by black arrows and negative regulation by red inhibitory arrows. Yellow nodes represent positive regulation by ethylene oxide (ETO) treatment for the model. Grey and yellow nodes represented genes of interest. Created in BioRender. Crouch, S. (2026) https://BioRender.com/muvn5po.

The senescence dataset^31^ was used to develop a one-shot model for senescence. All 12 genes of interest (ATM, CDK2, CDK4, CDKN1A, MAPK14, MTOR, NR1H4, PCNA, RB1, SPI1, TP53, ZFP36L1) were taken from previous iterative modeling (Fig. 3). A prompt was used to follow the pipeline structure based on the instruction, not to expand the gene list (Fig. S7, sup). In the next step, the GenAI request eliminated NR1H4 from the sgGRN due to the absence of regulation determination for NR1H4 in this case. Because the second prompt (Fig. S8, supplement) contained strict rules for determining interactions using GenAI, NR1H4 did not interact with any of the included genes. To reduce free parameters, two interactions were set to 0 (ATM—> RB1, 2 → 0; MTOR → CDKN1A, 2 → 0), as direct interactions were present here. The result is given in Fig. 4. The total Chi-2 value is 29.19 with 77 data points and 60 free parameters, and *p* = 0.0328, which is formally below the 5% threshold. To determine whether the subsequent steps are based on a stable, data-driven structure or whether the fit is primarily driven by irrelevant parameters that consume degrees of freedom without increasing explanatory power, 28 parameters with values below the threshold were fixed after L1 regularization. The purpose of this step was to define the most relevant parameters for the L1-regularized model. Then, a refit was performed on the reduced structure without regularization. The Chi-2 value of the fit remained virtually unchanged (before refit Chi-2 = 29.19, after refit Chi-2 = 29.16), even though the number of free parameters was significantly reduced (nParam = 32, dof = 45 (*p* = 0.968); before refit: nParam = 60, dof = 17 (*p* = 0.033)) and 7 of the 8 interactions considered were retained. These results suggest that the essential part of the model structure is robust and that the model remains useful as a working basis for further analysis. Accordingly, we perform the following analyses on the complete model and finally evaluate the goodness of fit and robustness consistently regarding the structure reduction since these eliminated parameters represent interactions that do not influence the model output.

A possible next step here could be iterative modeling as described above (Iterative modeling via GenAI) to incorporate additional genes, e.g., those on which drugs act, that we would like to investigate with respect to an optimal treatment or to further improve even genes that deviate a lot from the measured data. For this purpose, the orange star marks CDKN1A, which has the highest Chi-2 value in this model.

### Evaluation of GenAI

The subsequent evaluations were applied to the result model comprising 12 nodes obtained from iterative modeling. To determine whether GenAI outperforms simple estimation models and their interactions, we conducted a comparison with 10 random models. Table 1 illustrates the GenAI model’s success in fitting the data, yielding a final Chi-2 value of 18.765. We used a multi-start procedure to determine suitable initial values for the optimization process. Another reason for this decision was to ensure that the comparison was not unduly influenced by the choice of initial parameters. To this end, we deliberately refrained from using predefined initial values. Consequently, the AI-based model yielded a lower Chi-2 value compared to the model derived from the iterative final state (Fig. 3; Chi-2 = 33.51). A multi-start procedure identifies promising initial values for the fitting and then performs the parameter estimation based on these values. However, this approach is computationally much more intensive than a conventional fitting method based on a single predefined initial value. All random models exhibit a higher Chi-2_final value (Table 1) relative to the GenAI model. The quality of the model was evaluated by AIC, AICc, and BIC (lower values indicate a better fit, taking also the complexity of the model into account). The *p*-value evaluates the likelihood of the observed Chi-2 under the hypothesis of only white noise as model deviations from the data, given the assumed additive Gaussian error model and degrees of freedom. All criteria consistently favored the GenAI model (Table 1).

**Table 1:** GenAI model versus random model.

Mode	Chi 2_final	*p*_value	AIC	AICc	BIC
AI	18.765	0.927	128.765	348.765	262.460
r_001	30.806	0.375	140.806	360.806	274.501
r_002	58.593	0.001	168.593	388.593	302.288
r_003	32.020	0.319	142.020	362.020	275.715
r_004	60.320	0.001	170.320	390.320	304.014
r_005	81.382	0.000	191.382	411.382	325.076
r_006	449.675	0.000	559.675	779.675	693.370
r_007	2014.342	0.000	2124.342	2344.342	2258.037
r_008	62.844	0.000	172.844	392.844	306.538
r_009	103.662	0.000	213.662	433.662	347.357
r_010	252.777	0.000	362.777	582.777	496.472

The iterative model (Fig. 3) was employed to evaluate the GenAI model against a random model. The data points, free parameters, and number of nodes, along with their corresponding gene names, remained the same; only the interactions were altered, and a multi-start approach was employed to identify the optimal initialization for fitting.

AIC, Akaike Information Criterion; AICc, Corrected Akaike Information Criterion; BIC, Bayesian Information Criterion, and *p*_value.

A subsequent evaluation should assess the response to determine whether the GenAI yields consistent outcomes (Table 2). The test demonstrated that when the model was prompted to include three additional genes, it consistently identified Cyclin D1 (CCND1), E2F Transcription Factor 1 (E2F1), and Cyclin Dependent Kinase Inhibitor 2A (CDKN2A). In this study, temperature = 0 and top_*p* = 0 were set to ensure that the generation was as deterministic as possible and that the most probable tokens were prioritized in every case. In the API for controlling large language models, the temperature parameter governs the degree of random variability in token selection, while top_p (Nucleus Sampling) restricts the selection to those tokens whose cumulative probability exceeds a specified threshold.

**Table 2:** Evaluation of the generative AI output.

Request	GenAI response
1	ATM, CDK2, CDK4, CDKN1A, MAPK14, MTOR, NR1H4, PCNA, RB1, SPI1, TP53, ZFP36L1, **CCND1, E2F1, CDKN2A**
2	ATM, CDK2, CDK4, CDKN1A, MAPK14, MTOR, NR1H4, PCNA, RB1, SPI1, TP53, ZFP36L1, **CDKN2A, E2F1, CCND1**
2	ATM, CDK2, CDK4, CDKN1A, MAPK14, MTOR, NR1H4, PCNA, RB1, SPI1, TP53, ZFP36L1, **CDKN2A, E2F1, CCND1**
4	ATM, CDK2, CDK4, CDKN1A, MAPK14, MTOR, NR1H4, PCNA, RB1, SPI1, TP53, ZFP36L1, **CDKN2A, E2F1, CCND1**
5	ATM, CDK2, CDK4, CDKN1A, MAPK14, MTOR, NR1H4, PCNA, RB1, SPI1, TP53, ZFP36L1, **CCND1, E2F1, CDKN2A**
6	ATM, CDK2, CDK4, CDKN1A, MAPK14, MTOR, NR1H4, PCNA, RB1, SPI1, TP53, ZFP36L1, **CCND1, E2F1, CDKN2A**
7	ATM, CDK2, CDK4, CDKN1A, MAPK14, MTOR, NR1H4, PCNA, RB1, SPI1, TP53, ZFP36L1, **CCND1, E2F1, CDKN2A**
8	ATM, CDK2, CDK4, CDKN1A, MAPK14, MTOR, NR1H4, PCNA, RB1, SPI1, TP53, ZFP36L1, **CCND1, E2F1, CDKN2A**
9	ATM, CDK2, CDK4, CDKN1A, MAPK14, MTOR, NR1H4, PCNA, RB1, SPI1, TP53, ZFP36L1, **CCND1, E2F1, CDKN2A**
10	ATM, CDK2, CDK4, CDKN1A, MAPK14, MTOR, NR1H4, PCNA, RB1, SPI1, TP53, ZFP36L1, **CDKN2A, E2F1, CCND1**

GenAI was asked ten times which genes should be added to the network. 12 genes were taken over from the previous model generation (black), and 3 new genes were suggested by GenAI (bold). Setup: temperature = 0, top_*p* = 0 (prompt; Fig. S6 (supplement) was used).

Overview of approaches for generating GRNs with or without LLM-based methods.

To highlight the distinguishing features of DataXflowGen compared to existing approaches, a comparative analysis was conducted between our GenAI-supported workflow and established GRN methods, both with and without the use of LLMs (see Table 3). A thorough evaluation of existing methods revealed substantial variations in the implementation of language models across diverse approaches. The comparisons conducted using Table 3 demonstrated that LLM-based methods exhibit a broad spectrum of roles for language models.

**Table 3:** Comparison of reverse engineering methods for generating GRNs with or without LLMs.

Approach	Core idea	Who sets edges/nodes?	sdGRN strength	LLM use?
DataXflow-Gen	ODE-based sdGRN + optimal control; LLM suggests candidate regulators/to generate an initial model or interactions for poorly fitting genes (highest Chi^2^)in an iterative loop. ODE-based model can be directly tested via external stimuli framework (included in pipeline)	Edges: Mechanistic RE + Control decides: LLM specifies the edges Node: Expands nodes and their edges. LLM can suggest new regulators that are not yet included in the model; these will become new nodes along with their edges	Strong for dynamic, intervention-oriented sdGRNs grounded in quantitative fits and optimal-control analysis	LLM is used for literature-based gene/edge suggestions and pairwise regulation classification, not part of the fitting process. Data fitting step is used for evaluation
GRNPT^22^	NN/Transformer on single-cell trajectories with text-based gene/TF embeddings as prior	Edges: Data-driven NN, guided by LLM-derived priors; training on data decides final edges Nodes: Fixed node set from the scRNA/TF list; LLM does not dynamically add/remove genes, only shapes edge priors between existing nodes	Helpful for trajectory-guided sdGRNs when literature is abundant but data are limited	Uses domain text-LLMs to build gene/TF embeddings as priors inside the model; no chat-style LLM in the core method
LLM4GRN^21^	LLM directly scores gene pairs (activation/inhibition/none) from text	Edges: LLM is primary edge decider; Data that is only used retrospectively via GRouNdGAN, which merely checks whether a GRN proposed by LLM can generate synthetic scRNA-seq-like data (GAN-based simulation, no ODE or data-driven edge fitting) Nodes: Typically fixed candidate list (e.g., known TFs + variable genes); LLM may suggest extra TFs in text, but node set is usually chosen beforehand, not learned	Knowledge-driven sdGRNs; strong with rich literature, weaker for novel/understudied biology	Uses a large language model (GPT-class) directly for causal edge inference via prompting and iterative prompt refinement
Classical static RE^46–49^	Correlation/MI, LASSO, GENIE3	Edges: Purely data-driven statistics/ML on expression; edges inferred among preselected genes/TFs Nodes: Fixed node set defined by preprocessing (e.g., HVGs, TF list); algorithms do not add/remove genes during training	Good for state-specific GRNs via stratification, but it does not capture the dynamic evolution of gene regulation over time or the transitions between states	No integrated LLM
Dynamic RE^23,36,37,50^	DataXflow, dynGENIE3, GRIT, and other ODE-based GRN models	Edges: Mechanistic fit of edges/parameters to time series or pseudotime Nodes: Fixed node set chosen by the modeler (subset of genes/TFs); structure learning changes edges, not which genes exist	Strong for continuous sdGRNs over time/conditions; highly interpretable parameters and feedback loops	No integrated LLM
Deep-generative RE (diffusion /informative priors)	DigNet^39^: diffusion-based generative model; InfoSEM^40^: deep generative model that performs GRN inference	Edges: both infer edges in a data-driven way inside a generative model; DigNet via a multi-step diffusion process, InfoSEM via variational inference with structural priors Nodes: fixed gene/TF set from the input data in both cases, no dynamic node expansion	strong for cell-type-specific GRNs from scRNA-seq, especially when one wants global network architecture (DigNet) or to exploit strong biological priors (InfoSEM)	No integrated LLM

RE, Reverse engineering; NN, Neural networks; TF, Transcription factor; ODE, Ordinary differential equation; sdGRN, Signed gene regulatory network; LLM, Large language models; HVGs, Highly variable genes; ML, Machine learning.

For example, a novel Transformer‐based framework, called GRNPT, uses LLM-derived information about text-based embeddings as prior information in the neural network, while LLM4GRN (Discovering Causal Gene Regulatory Networks with LLMs) derives its edges largely directly from LLM decisions^21,22^. Compared with DataXflowGen, which utilizes LLMs for GRN construction similar to GRNPT and LLM4GRN^21,22^, DataXflowGen uses classic ODE-based models to evaluate the hypothesis generated based on the knowledge of the LLM and the retrieved references processed by the LLM with data of the concrete scenario. DataXflowGen uses the data to validate the hypothesis from the AI assistant rather than for learning the GRN, since we assume that a lot of the information for generating GRNs should be available. Furthermore, such a feedback loop opens the door to an agentic workflow that constructs the GRN that fits certain situations and thus infuses the context of the concrete scenario into the reference search of the AI agent. The iterative alteration between hypothesis generation and testing (model fitting) allows the agent a step-by-step, educated trial-and-error procedure to test several ideas in cases where the concrete regulation is not clear from the current state of the network, given data and context. By allowing iterations, we free a method from the burden of finishing in one shot and gain some experience in a particular situation to see which of the knowledge from similar situations might help best. While classic static and dynamic Reverse Engineering (RE) methods (e.g., GENIE3/dynGENIE3, GRIT) infer the edge structure completely data-driven within a predefined node set^36–38^, DataXflowGen allows the dynamic expansion of node sets and their edges based on an iterative LLM-supported literature search and thus allows an alternative way to construct a GRN that is validated via an ODE system that is fit to the concrete data. This method has advantages in cases where the time series data does not allow causal reconstruction well, e.g., due to a lack of quality, and thus causal information can be infused via the LLM and reference search that can then be at least validated with the data. However, both methods can be combined by providing an initial GRN to DataXflowGen that is then refined with the iterative improvement of the network, where genes deviate most from their data. Another concept to combine the methods is that dynGENIE3 or GRIT can be used to make an initial hypothesis for that part of the network that does not fit well to the data. For example, after several iterations, where our proposed framework does not provide a fitting solution due to, e.g., the fact that a new effect is discovered that is not sufficiently described in existing literature. However, we have to keep in mind that in contrast to the other part of the network, the part learned from the data might require additional validation data to make sure the suggested new GRN part really describes a fitting (ground truth) regulation and no overfitting to the data, where the suggested GRN part might even rather be a correlation on the specific dataset than a causality. DataXflowGen completely separates quantitative validation (ODE model + optimal control) and qualitative knowledge (LLM-based suggestions), which allows the full usage of existing knowledge and the full data for thorough validation. The qualitative suggestions are meaningful for obtaining regulation and genes that are important in a certain context, e.g., that the model is supposed to model lung cancer and that certain drugs are supposed to be administered. Thus, the model can be kept small and focused on the most important genes, and the data can be selected so that a network for the full dataset might not be required. On the other hand, data collection can be focused on the most important genes in a certain context and use budget rather for more experiments where external stimuli are applied to get more insight into how genes regulate each other by intervention. DigNet^39^ and InfoSEM^40^ are deep generative reverse engineering methods that derive GRNs directly from expression data. DigNet uses diffusion models, while InfoSEM uses variation inference with informative structural priors^39,40^. Both methods use a fixed set of genes/transcription factors (TFs) and are purely data-driven, in contrast to DataXflowGen’s LLM-assisted node and edge expansion that can infuse the latest publications from the web since it utilizes AI assistants such as Perplexity^29^, into the modeling process. This makes DataXflowGen particularly well-suited for sdGRNs that are dynamic and intervention-oriented, where both accurate temporal dynamics and clear, literature-based extensions of the network topology are important. However, as mentioned above, tools like DigNet or InfoSEM can be used to analyze parts of the graph from the data where there is insufficient current knowledge. Summarizing, we remark that in whatever way a GRN is constructed, be it by an AI assistant or by ML models that analyze time series data, the corresponding hypothesis should be checked on data that has not been used for the GRN construction. This work proposes a system of ODEs for validating, as it usually has much fewer free parameters than ML models, which might be fit during the GRN construction and tend to overfit. By utilizing a system of ODEs for validation with a relatively small number of free parameters, use cases where data is more expensive can be approached. Moreover, once an ODE system fits the data well, it immediately enables further investigations of how best to steer the network by influencing it with external stimuli (where a mathematical model of how to include external stimuli into GRNs is explained e.g. in^41,42^), such as drugs, to achieve a desired state, such as a therapy to fight cancer cells and steer them to apoptosis^43–45^, which is another feature of DataXflow [^23^, 2.6. External Stimuli Framework]. Thus, the information encoded in the network can be exploited for investigations of how to influence the network best to solve an associated research problem. In turn, these investigations are an additional way of generating a test to check if the network describes the regulation in a certain scenario well by applying the corresponding external stimuli and seeing if the hypothesized effect of the external stimuli can be observed, which is that the real system is in the desired state. If not, the experiment has already generated a new dataset that can be included in the model fitting and another iterative adaptation round where the AI assistant has to come up with a hypothesis that is supposed to change the model such that it fits the updated data better.

In summary, these comparisons demonstrate that DataXflowGen represents a complementary niche between purely data-driven and purely LLM-driven GRN generation, providing a mechanistic baseline that can both exploit existing knowledge and rigorously validate new hypotheses on experimental data.

## Discussion

Our work shows that using DataXflow^23^ with LLM-based GenAI provides a more efficient approach to generating signed gene regulatory networks (sgGRNs) from gene expression data. Integrating GenAI into our pipeline will significantly advance personalized medicine and optimized therapy. Furthermore, its applications are not limited to research on cancer or the aging process in human subjects.

### Mathematical modeling for GRNs by GenAI

The integration of mathematical modeling with current generative AI (GenAI) technologies has the capacity to simplify and enhance the development of sgGRNs while maintaining biological plausibility. In this context, various methods have been established to obtain GRNs from sequencing data as graphs or dynamic models. In addition to our DataXflow framework^23^, these include approaches such as DigNet by Wang and Liu, a discrete diffusion-based generative model that performs a multi-stage denoising process in graph space to develop cell-type-specific GRNs from single-cell RNA sequencing data (scRNA-seq), thereby effectively restoring regulatory structures despite noise and sparsity of data^39^. Cui et al. offered InfoSEM, a deep generative variational Bayesian model that employs informative priors from biomedical text embeddings and established interactions to construct GRNs predominantly in an unsupervised approach, thereby enhancing the prediction of interactions among previously uncharacterized genes in particular^40^. In contrast, GRNPT and LLM4GRN are two methods that utilize LLMs but differ in their approaches. GRNPT uses data-driven neural network inference guided by LLM-derived priors to determine interactions^22^. In LLM4GRN, the LLM is mostly used to make decisions on the edges^21^. This study examines how the use of LLM-based GenAI as a tool for literature research and model design can improve the rapid development and improvement of biologically relevant GRN models within our DataXflow pipeline. The pipeline, now called DataXflowGen, uses LLM not only to extend the edges but also to incorporate nodes that improve and expand the model in a sequential way that allows trying several ideas from similar situations to see which one fits best.

In addition to classical differential-equation-based and mechanistic models, physics-informed neural networks (PINNs) have emerged as a prominent approach in scientific machine learning. Emmert-Streib et al. demonstrate the efficacy of integrating the underlying differential equations describing the dynamics directly into deep learning models to solve systems of differential equations, yielding data-driven yet physically consistent solutions^51^. The present work conceptually aligns with the current approach, as the DataXflowGen pipeline also depends on differential-equation-based modeling. It focuses on the generation of interpretable mechanistic GRN models, which are analogous to the ODEs describing physical scenarios, and can be integrated for predictions relying on knowing the corresponding law of interactions, which offers an alternative to purely black-box architectures.

### Inside the iterative senescence model

Biological interpretation of GRNs is increasingly utilized in research and medicine to transition from single-gene analysis to a comprehensive understanding of disease mechanisms, enabling the identification of critical regulators, biomarkers, and potential therapeutic targets in complex diseases, such as cancer^45,52–55^. Clinical and translational research demonstrates that GRNs, including patient-specific networks, enhance survival prognosis and disease stratification while uncovering novel regulatory mechanisms that are not detectable by conventional differential expression analysis alone^54,56^. The previous version of DataXflow has already demonstrated the validation of sgGRNs and the precise identification of targets afterwards, specifically for non-small cell lung cancer^23^. This part of the pipeline is also incorporated in our extended pipeline, DataXflowGen.

Here, we developed an iteratively generated senescence model containing 12 genes (nodes) (Fig. 3) from a senescence study by Wechter et al.^31^. The gene CDKN1A, stimulated by ethylene oxide (ETO), was chosen as the initial focus^31^. The newly included genes CDK2, CDK4, RB1, TP53, PCNA, ATM, ZFP36L1, SPI1, NR1H4, MTOR, and MAPK14 are closely associated with cellular senescence and age-related signaling pathways, with several genes well described in the literature regarding the p53–p21–RB axis^57,58^. CDKN1A serves as an essential CDK inhibitor, inhibiting CDK2/CDK4 after p53 activation, thereby keeping RB1 in a hypophosphorylated state. These induce a sustained cell cycle arrest associated with cellular senescence across multiple pathways^59,60^. RB1 is a key factor in senescence-associated arrest and tumor suppression^61^, whereas alterations in PCNA expression and activity are intimately linked to proliferative potential and age-related tissue modifications^62,63^. ATM is an important kinase in the DNA damage response and promotes irreversible cell cycle arrest and the production of the senescence-associated secretory phenotype (SASP) by activating p53/p21 and NF-κB. Thus, ATM signals play a crucial role in DNA damage-induced senescence^64–66^. ZFP36L1 regulates the stability of inflammation-associated mRNAs, and its phosphorylation, dependent on mTOR/p38 signaling pathways, affects SASP and consequently the phenotype of senescent cells^67^. SPI1 is a hematopoietic transcription factor that regulates differentiation, inflammatory pathways, and senescence-associated transcription programs in myeloid and lymphoid cells, with its function being significantly cell type-specific^68^. MTOR and MAPK14 (p38α) are components of well-established signaling pathways in senescence. Both promote the onset and persistence of the SASP dependent upon the environment, and their pharmacological suppression may reduce senescence-related alterations^67,69,70^. NR1H4 (FXR) is primarily characterized as a nuclear receptor involved in the regulation of bile acid and metabolism. Although there is evidence that it is involved in organ aging and inflammatory processes, the direct and canonical link to senescence remains unclear in the literature and likely varies depending on the context and tissue type^71–73^. All genes, except NR1H4, that were integrated into the GRN based on the response of the LLM-based GenAI ‘PerplexityAI’^29^ are associated with senescence. Although this compact network facilitates mechanistic interpretation, it may not capture the full complexity of senescence regulation.

A further significant limitation of this study is the absence of independent validation using external datasets or biological experiments. While the proposed framework is evaluated based on internal model consistency and goodness-of-fit metrics, no validation was performed using independent patient cohorts, publicly available transcriptome datasets, or experimental approaches such as quantitative PCR (qPCR). Consequently, the generalizability and robustness of the regulatory mechanisms derived must be confirmed. Hence, the clinical relevance of the presented results should be interpreted with caution, and future work should focus on validating the identified regulatory interactions across independent data sources and experimental systems. The focus of this work is the augmentation of the modeling process with AI assistants and the integration of the corresponding APIs into the existing DataXflow framework.

### Strengths and limitations of GenAI-based sgGRN generation

The application of GenAI generating models in healthcare settings involves numerous major limitations and potential risks. Templin et al. identified six principal problems for GenAI in digital health, including bias, data protection and privacy, misconceptions, hallucinations, and clinical dangers stemming from usage or overconfidence in GenAI outcomes^74^. Tung et al. identified further ethical and practical challenges, such as insufficient transparency and explainability, ambiguous legal responsibilities, and deficiencies in governance^75^. Our approach produces a GRN that is understandable by humans and can be tested on concrete data, which mitigates the explainability and hallucination issue. Concurrently, Currie et al. emphasized biases related to gender and ethnicity in text- and image-based GenAI and the consequences for medical imaging^76^. Recent systematic reviews and meta-analyses also show that the diagnostic performance of GenAI systems in healthcare varies considerably depending on the clinical task and setting, and generally remains below the level of experienced human experts in complex, real-world scenarios^16,77^. Moreover, these studies demonstrate restricted generalizability across institutions and patient demographics, alongside significant heterogeneity and methodological deficiencies in the foundational evaluation studies, collectively constraining the reliability of current GenAI tools for clinical application^16,27,74,78,79^.

This approach considers the inherent limitations of GenAI. It is used exclusively to assist in selecting and justifying individual nodes and edges in GRNs rather than for unrestricted network development. Carefully incorporating individual genes (nodes) into the network and systematically querying and manually verifying specific gene–gene interactions reduces the likelihood of hallucinations and systematic misclassifications while maintaining control through domain-specific expertise. Furthermore, integrating the resulting GRNs into the DataXflowGen pipeline provides an additional layer of validation. The validation is performed as this pipeline includes quantitative calibration and testing through D2D^30^-based parameter fitting and model evaluation to confirm that the GenAI-supported network structures are consistent with the experimental data and possible system dynamics.

In our research, we used PerplexityAI^29^ because of its cost-effectiveness. However, our pipeline is also compatible with other GenAI systems, such as those from Gemini^80^, Claude^81^, and OpenAI^82^, which offer API access. We need such AI assistants to provide us with hypotheses. Since we instruct the AI assistant to output in a certain format, the replacement of one AI assistant by another can be easily done. A study conducted by Pan et al. evaluated four different GenAI chatbots and showed that Perplexity performed well in providing accurate and reliable medical information for various types of cancer^83^.

For some reason, the limitations are particularly evident in the variability of responses regarding edges. These responses vary even when the ‘temperature’ parameter is set to zero. This was also described in the study by Alzarea et al.^84^. They found that PerplexityAI and Meta AI changed their recommendations for antibiotics over time based on the same case^84^. A study by Yuan et al.^85^ offers a preliminary systematic explanation for why the supposedly deterministic decoding in LLMs can lead to different results. They attribute the non-determinism in greedy decoding to the non-associativity of floating-point integers and hardware- and batch-dependent rounding effects, which impact the output of the highly non-linear functions that represent LLMs, and reveal significant differences in the accuracy of conclusions as well as response length across different GPU and precision configurations. This research emphasizes that the reproducibility of benchmarks across commercial chat APIs is challenging and explains the phenomenon that identical inputs at a temperature of 0 lead to different generations when numerical conditions fluctuate at the system level^85^. Since we might mostly not be able to control the implementation of AI assistants, and thus we might need to deal with the non-reproducibility of the GRN generation, it is even more important that DataXflowGen has a systematic way to test the AI assistant’s suggestions. Even if not reproducible, if a GRN cannot be rejected to fit the data, we have a working candidate of a GRN that cannot be rejected based on data, which is already beneficial. Furthermore, by analyzing the fit of each gene in the network, we can easily see issues of potential former suggestions in case they were based on an unfortunate run of an API. Since these AI assistants usually retrieve sources for answering, if we get access to them, we can also manually check the references that the AI assistant consulted for gene regulations whose correctness we doubt. Human effort is always directed to where issues are left, while the working parts from the AI suggestions can simply be used.

The advantages of omics-based large language models for target and network identification, as outlined in “Omics-based LLMs: A New Engine for Drug Discovery Innovation”, by Sheng et al.^79^, are limited by the fact that these models should be regarded exclusively as hypothesis-generating instruments. This is due to restricted ground truth, data bias, and the absence of prospective validation^79^. Consistent with this recommendation, LLM-based GenAI served as a tool for hypothesis generation in our research and was evaluated and improved based on “goodness of fit” (D2D^23,30^ step in our new pipeline). The advantage of such large models is that they can be trained on a large corpus of data and can make a prediction in a scenario where there is no or only a small amount of data. In case the current scenario is similar to one of the training sets, the prediction might be helpful, and therefore, such large models are a cheap way to get ideas about what might solve a research question without their own data generation. They can transfer information from a similar situation to the current one and thus find and reuse fitting information. However, in cases where the prediction does not work, we need a systematic way to improve. While a suggestion from a black-box model does not provide any guiding principle, an ODE system generated from a GRN can be analyzed, and the parts that do not fit well in a certain situation can be systematically improved by analyzing the parts that fail.

Our sgGRNs, which are based on ODEs, are well interpretable, since each node represents a gene, each edge represents a specific regulatory influence (activation or inhibition), and each parameter represents a biologically meaningful quantity, such as the strength of an interaction. This allows us to reason mechanistically about cause-and-effect relationships within the network, for example, by asking how a therapy might alter the regulation of certain genes. In contrast, many AI models behave like black boxes. While they can fit the data well, it is often unclear which internal components encode which biological mechanisms, requiring additional methods for interpretability. For example, Pfeifer et al. propose counterfactual paths that show how a prediction would change if certain input features were modified. This makes it easier to identify which variables determine the decisions of a trained black-box AI model and to visualize these dependencies in a form that is understandable to humans^86^.

In summary, LLM-based approaches to GRN development must be validated with particular care, as they inherit the above-mentioned fundamental limitations, and their predictions should therefore be consistently verified against current literature, expert knowledge, and experimental evidence^27^, in particular in cases where a wrong prediction is costly, such as an incorrect therapy, particularly an incorrect drug combination. Another approach could be to first query a large language model to suggest context-specific candidate genes, and then test these candidates in silico, for example, using our ODE framework^87^.

### Data limitations and challenges for gene regulatory networks

The construction of GRNs is subject to data-driven limitations^88^. For example, single-cell RNA sequencing (scRNA-seq) data is sparse, with gaps that cause active genes to appear inactive (dropouts). This can lead to genuine regulatory relationships being confused with technical noise^89,90^. Additionally, sdGRNs are typically only available based on a few experimental conditions, short time courses, and heterogeneous cell populations. This makes it extremely difficult to identify stable, causally interpretable edges due to batch effects, pseudotime instability, and insufficient perturbation data. Consequently, many potentially relevant regulators remain “hidden” in the noise of the data^91–97^. The Bulk RNA-Seq method averages expression across all cells in a sample, resulting in robust, easily reproducible signals, but at the expense of cell state resolution. Differences in the proportions of cell types can appear as ‘regulation’ in bulk and thus systematically distort coexpression/network edges^98^. scRNA-seq can reveal state- or cell type-specific programs by separating this heterogeneity, but it is more strongly influenced by technical effects and sparse counts. To make reliable group comparisons, it is crucial to correctly assign biological replicates. Otherwise, many false positives may arise. This is why aggregated ‘pseudobulk’ analyses are often more stable^99^. We remark that a GRN can only be built for one cell type, and an analysis needs to be done if the differences in the cell population come from an exposure to different external stimuli that make the same GRN exhibit different values or if there are different ground truth GRNs, such as in a heterogeneous tumor. When ensuring a homogeneous external stimuli environment, differences in the GRN can be analyzed as in [^23^, 4.2. Modeling heterogeneous tumors], where a GRN can be constructed for each cell population, and we might not necessarily have to start from scratch by analyzing differences between the populations. However, since repetitions are needed to obtain an expected error for the validation (best values and errors for each time point), a clear and stable identification of each cell population is needed. However, for cell annotation, there are also techniques available; please see^100–103^.

The ability to create models with GenAI is a fast method of model development. Based on our pipeline, which evaluates the model based on the goodness of fit, correct data collection plays an important role. In research studies, it is important to consider in advance what question we want to answer. As early as 1994, P E Plsek wrote a multi-part tutorial covering important aspects of data collection that are still relevant today^104^.

One requirement to apply DataXflowGen to its full potential is the availability of data of a specific kind. In order to validate the GRN via a system of ODEs, we need the best values and errors of these best values or uncertainties, respectively, to use the best-parameter-fit routine and decide if a model cannot be rejected based on statistics. Consequently, we need to repeat the experiments several times to see how the expression values for each gene and time point differ. From the corresponding standard deviations, we can calculate the standard error for the best value. We need to make sure that this error is a good measure of how the best value would change upon a repetition of the experiment. We would like to remark that even for a single cell measurement, where each cell can be seen as a repetition of the experiment, the corresponding (small) standard error might be too small, as a repetition of the whole experiment (several biological replicas) might lead to a shift of the whole population, which will enlarge the corresponding errors. So even in the scenario of single-cell measurements, repetition of the whole experiment might be required. GRNs are supposed to model causal relations between genes. To make our dataset powerful to falsify AI hypotheses that contain correlations, which might hold in certain scenarios and thus cannot be rejected, and leave only real causal relations, we need a dataset that includes the expression data under many different external stimuli. The rationale is that correlations might hold in certain situations but not in all. As an example, take a gene that influences two genes in parallel downstream. However, we can only analyze that the two downstream genes are correlated in the first situation and not causally connected in the second situation if we perturb these genes separately. Without such a dataset, no framework, be it based on ODE systems or ML models, can differentiate models that only map correlations or represent causal relationships. The more different experiments are done, the higher the quality in terms of causal relations we can expect from the model. However, a GRN can be used to plan the application of the external stimuli efficiently if we know which genes the external stimuli act on. Namely, we can see which parts of the network are not affected by external stimuli so far and where to apply further external stimuli to close gaps and not spend resources where a GRN would be redundantly tested. Please keep in mind that each experiment (different settings of external stimuli, e.g., combination or strength) needs to be repeated. In case there is a homogeneous cell population or the population can be separated into homogeneous ones, a bulk measurement technique might lower the costs such that the resources can rather be invested into the above-described data generation instead of single cell measurement. For more details about data generation suited for best-parameter-fit, please see [^23^, 4.1 Data generation optimized to apply DataXflow]. Consequently, we recommend a clear definition of the research goal, which defines what methods are needed to analyze it, which then defines the data needed to achieve the goal. If the properties of the problem are not clear at the beginning, such as if the cell population is homogeneous, it makes sense to investigate such subproblems step-by-step and allocate resources based on the needs of solving the subproblem. Currently, it is challenging to find datasets with the above properties, and we intend to mitigate this issue by providing a clear description of the required data above.

After a model is fit, there are further questions, such as whether the model is too sensitive to the data, which can be analyzed as suggested in Breitenbach and Dandekar^105^, or if the model deviations from the data are not improvable anymore, given the data, or there is a systematic error as addressed in [^106^, Conclusion].

The combination of complementary data science methods represents a promising approach for generating and validating data-driven hypotheses in complex biological systems^107^. For example, the integration of pseudotime analyses with GRNs allows for the linking of dynamic cell state trajectories with underlying regulatory mechanisms. Pseudotime methods facilitate the reconstruction of continuous developmental processes from single-cell data and the identification of temporally ordered changes in gene expression. GRN inference methods model causal or at least directed regulatory relationships between genes. By selecting genes whose expression dynamics vary significantly along the pseudotime, a reduced, biologically relevant subnetwork can be constructed that mechanistically reflects the observed temporal development^107^. Hypotheses can be formulated, for example, that an external stimulus, such as a drug (Drug X), induces a specific differentiation or reprogramming process. These hypotheses can then be tested to determine whether the inferred regulatory interactions consistently explain these dynamics.

In addition, the framework can be further developed to adopt an agent-based approach. In an initial stage, such an agent could autonomously explore datasets, identify structural patterns (e.g., trajectories, clusters, transition states), and identify the genes that carry important information separating the patterns with tools and frameworks developed as in, e.g.,^32–34^. Based on these information-carrying genes, an initial hypothesis can be derived about the underlying biological processes and relevant regulatory genes, including genes mediating drug effects, which could be incorporated. These hypotheses would then serve as guidelines for targeted GRN inference, with the agent determining which genes, signaling pathways, or interactions should be investigated as a priority. It is hypothesized that through an iterative loop of hypothesis generation, model-based validation, and, if necessary, another iteration of data analysis under the insights from the previous steps, an adaptive system could emerge that not only tests existing biological assumptions but also generates and refines new, data-driven hypotheses. Such an approach would extend the classical pipeline of analysis and interpretation toward a semi-autonomous, hypothesis-driven discovery system. The generated hypothesis can then be tested as explained in this work to finally come up with an explanation of the data.

In the context of treatment response, such an agent could also utilize specialized analytical tools to systematically identify candidate genes for specific phenotypes, such as treatment resistance, as demonstrated in the work on mathematical strategies for predicting resistant subpopulations based on single-cell data^108^. Building methods for identifying resistance-associated genes from bulk or single-cell transcriptomic data, the agent would first prioritize genes strongly associated with the observed resistance behavior and then use these as nodes for the nucleus of a GRN. Before the construction of the GRN, additional data would be collected for the genes most strongly associated with resistance if not already available. Next, in such a multi-stage process supported by an AI assistant, the network is analyzed for validation and iterative refinement of its structure if necessary. This results in a multi-step process. An initial pre-analysis selects genes most relevant to the phenotype of interest, while the agent’s domain knowledge (e.g., known signaling pathways, interaction partners, or drug targets associated with these genes) is used to enrich the GRN with biologically meaningful genes.

This is particularly relevant in the context of oncological applications, where the agent has the capacity to establish networks that not only elucidate the observed variations in the data but also identify genes targeted by approved or experimental drugs. Consequently, this potentially facilitates downstream analyses focused on modifying or preventing resistance mechanisms.

As future research, we aim to further automate the steps in generating GRNs. An important next step is to find ways to include the feedback after the fitting process into the AI assistant, such that it can identify where improvements are necessary and also provide tools/heuristics (see supplement ‘Subgraph analysis of non-fitting genes’) to analyze the network to start with the most promising candidates for the improvement. Furthermore, the AI assistant should detect when the fitting tool has stably converged and restart with a different set of parameters if convergence has not yet been reached.

In addition, we might need to extend the framework with more differentiated prompts if there are multi-experimental settings where, in different experiments, different external stimuli are applied. Then we need prompts specifying that under condition X, gene A needs activation, while under condition Y, gene A needs inhibition, and ask what genes good candidates might be to do so, for example, in treatment response scenarios where resistance and sensitivity emerge under different perturbations. This development represents a logical progression toward the establishment of an agentic, semi-autonomous GRN design and analysis framework.

## Conclusion

DataXflowGen represents a significant advancement in the automated and rapid development of sgGRNs, leveraging GenAI technology based on LLMs. While offering the same ease of use as its previous version, DataXflowGen provides substantially improved automation and flexibility in model generation. The pipeline is available via a public Git repository and provides transparent, reproducible scripts, as well as comprehensive LLM-based GenAI modeling documentation. Future work will focus on systematically evaluating DataXflowGen on diverse datasets and integrating DataXflowGen into broader systems biology and agentic workflows.

## Methods

**Fig. 5:**
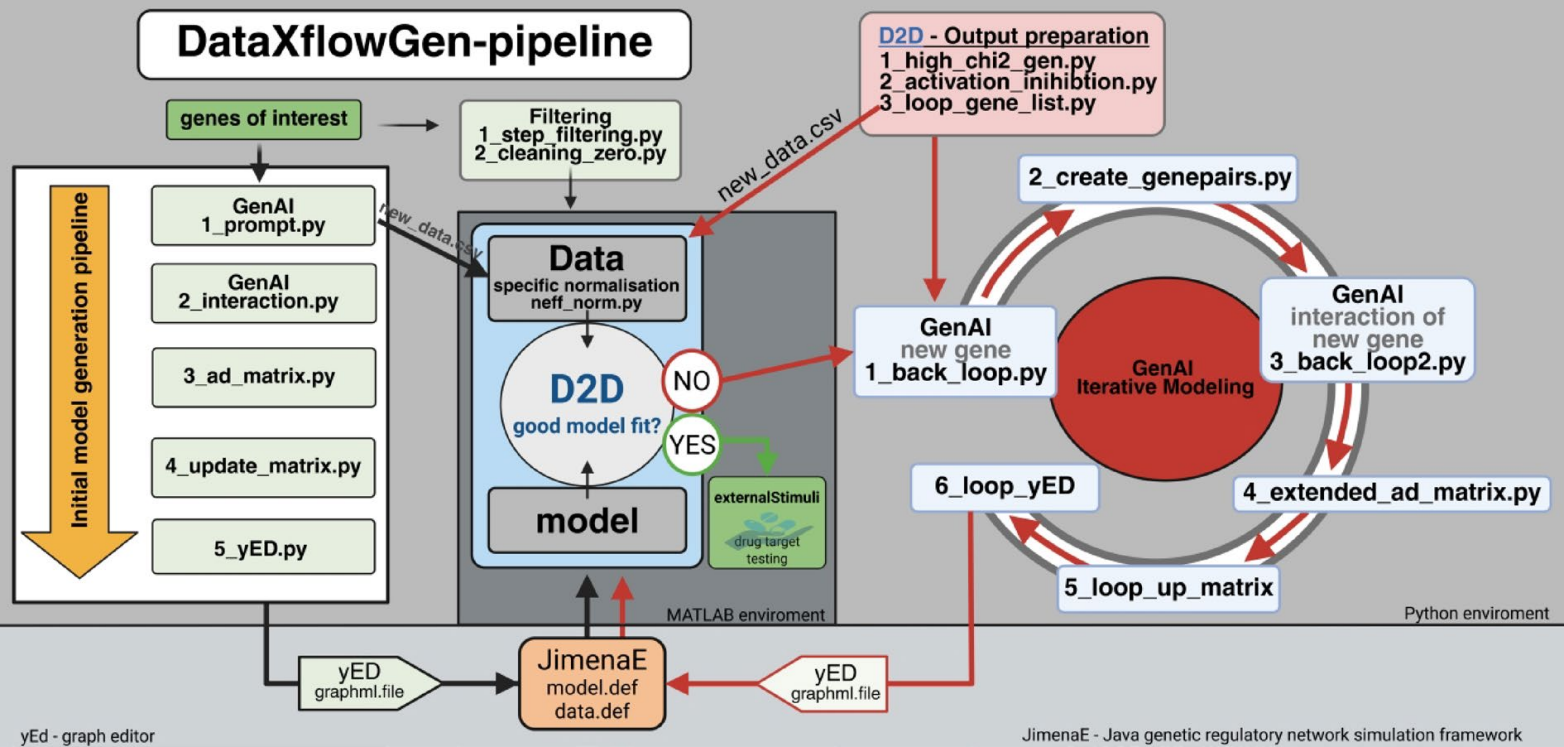
Overview of all steps of model generation via DataXflowGen. All steps are clearly outlined in the “Methods” section. All scripts and important files can be found here https://github.com/Sunbio1/DataXflowGen. Created in BioRender. Crouch, S. (2026) https://BioRender.com/908fj5z.

Our methodology combines GenAI-driven hypothesis generation with deterministic network modeling and iterative refinement. First, we utilize previous analyses (e.g., DEG or ML-based feature selection) and prompt GenAI to identify additional candidate genes and interactions, thereby expanding a small set of interesting genes into a broader pool of regulators and target genes. Second, we convert the GenAI output into a signed interaction network by querying gene pairs individually, encoding the results in a weighted adjacency matrix, and removing redundant indirect edges. Third, we translate this network into an ODE-based model within the Data2Dynamics framework, fit the parameters to time-resolved experimental data, and use regularization to obtain an interpretable network. An iterative feedback loop follows this. Genes with poor fit are identified (Chi-2), GenAI is prompted again to suggest upstream regulators specifically for these nodes that do not fit well, and the expanded network is refitted. This process continues until a stable, data-consistent GRN is obtained. In the following sections, we describe each of these steps in detail, from GenAI-assisted network generation through ODE-based model fitting to the refinement by the feedback loop (see Fig. 5 for a schematic overview).

The git repository contains all scripts and files and can be found here: https://github.com/Sunbio1/DataXflowGen.

### Data collection

Datasets may provide diverse information generated through various methods. On one side, significantly differentially expressed genes (DEGs) can serve as a basis for model development. Machine learning algorithms that identify significant distinctions between, for instance, young and elderly or sick and healthy persons are also predicated on genes and can be used for model development^32–34,108^. We chose an established dataset from senescence research here. This data originated with fibroblast cells exposed to Ethylene Oxide (ETO) treatment. A scRNA-seq analysis was conducted for seven distinct time points. We adopted the count tables from their analysis for our investigation^31^.

### Data preparation

Seven datasets were extracted from ‘.h5ad’ files (GSM7068361, GSM7068362, GSM7068363, GSM7068364, GSM7068365, GSM7068366, and GSM7068354) and filtered to retain cells with a minimum of 200 identified genes and genes expressed in at least 3 cells. Quality control measures were calculated and presented, including total count, detected gene quantity, and the ratios of mitochondrial (MT) and ribosomal (RPS/RPL) counts. The counts were normalized to a total library size of 30,000 counts per cell (normalize_total), and the normalized expression matrix was exported as a CSV file (‘1_step_filtering.py’). Filtering out rows with only zeros was performed (‘2_cleaning_zero.py’) and served as ‘GSMxxx_count_cleaned.csv’.

### Data-specific normalization

Data from each time point was taken from the cleaned count tables and limited to the genes specified in either ‘gene_list.csv’ or ‘loop_gene_list.csv’. The expression values were converted to a numeric format, and non-finite entries (NaN/ ± Inf) were removed. Negative values were excluded, but zeros were retained. Based on the data, it is important to evaluate whether zeros need to be filtered out. Too many zeros in scRNA data can cause problems during specific normalization (see also the Discussion on data issues ‘Data limitations and challenges for gene regulatory networks’). The values were subjected to a log transformation using log(1 + x) = log1p(x). The maximum log-transformed value was calculated for each gene and time point, and all log-transformed data were normalized per gene over all points in time by this maximum. This resulted in values within the range [0, 1]. The mean of the normalized results was determined for each gene and time point. The effective standard error was reported as SE_eff_ = SD/√(N_eff_). For our dataset, we used an effective sample size of N_eff_ = 15 to obtain conservative uncertainty estimates (i.e., larger effective error bars / standard errors). This choice reflects that individual cells from the same single-cell experiment are not independent biological replicates and that cell-to-cell variability within one sample does not capture the full uncertainty across experiments or populations. The specific value N_eff_ = 15 was selected empirically because it resulted in error bars of an acceptable magnitude while avoiding the unrealistically small standard errors that occur when all cells are treated as independent replicates. The resulting summary table for each time point (mean and SE_eff_ per gene) is saved as ‘neu_data_log_15neff.csv’. The script ‘neff_norm.py’ can be found in the ‘normalization’ folder here https://github.com/Sunbio1/DataXflowGen.

### Model generation pipeline

We have developed an iterative workflow that combines GenAI-based knowledge extraction with deterministic network construction and model refinement. Starting with an initial list of genes, GenAI is first used to expand the set of candidate genes to include additional, functionally related genes. Subsequently, GenAI is queried in a controlled, pairwise manner to assign signed interaction types (activation or inhibition, direct or indirect) between selected gene pairs, which are encoded in a weighted adjacency matrix. This interaction graph is then translated into a mechanistic model within the D2D framework, where model parameters are fitted to time-resolved experimental data and regularized to obtain well-identified network structures. Based on goodness-of-fit evaluations, poorly fitted genes and sensitive parameters are identified and used to define targeted feedback loops. GenAI is then queried again to suggest plausible upstream regulators, the interaction network and adjacency matrix are expanded, and the cycle of fitting and evaluation is repeated until a stable, data-consistent network is obtained. Figure 5 provides a general overview of this methodology and its implementation, mapping each conceptual step of the pipeline to the corresponding analysis scripts used for data preprocessing, the GenAI-based query, model construction, parameter estimation, and iterative network refinement. The individual steps and the scripts used are explained in detail below.

### Initial model generation pipeline

The goal of this first phase is to compile a biologically plausible set of genes and potential interactions that will later form the backbone of the GRN. We use GenAI to expand an initial list of genes of interest to include additional, context-specific regulators and interaction partners based on literature references and previous analyses before translating these suggestions into a structured network representation.

### GenAI response to find possible new interaction genes

In this first step, GenAI acts as a knowledge-based assistant that suggests additional genes likely to interact with an initial set of genes of interest within a specific biological context (e.g., tissue, phenotype, treatment).

In our pipeline, a prompt (1_prompt.py, Fig. S6 in Supplementary Material (sup)) was run via API access to PerplexityAI^29^ to create a gene list of possible interaction partners by using genes of interest identified through previous analyses, like DEG analysis or ML approaches. Such ML approaches can be used to identify genes with high predictive power for separating phenotypes based on expression values^32–34,108^. In a prompt, we can provide context that the following genes have been analyzed to have a high predictive power and we would like to have the most relevant ones from them to build a causal regulatory network to model the differences between the corresponding phenotypes as well as let the AI assistant add more nodes in case we would also like to model genes that interact with drugs and integrate their impact into the network.

Besides the list of important genes from a data analysis, we provide further context in a prompt from which tissue the cells are taken and for what purpose we would like to use the model for. Below, we provide the used prompt (Figs. S6, S7, and S8 in sup). In our case, we used CDKN1A as a starting point, an important gene for studying senescence^31^. The ‘sonar’ LLM model was used via API, which is supported by PerplexityAI^29^. The API query was performed using Python (version 3.13.1) and the package openai (version 1.59.5). To get a deterministic output, the temperature was adjusted to 0, and to confine the generative AI response to highly probable solutions, top_p was set to 0. These settings are implemented for each API request. API response was saved to ‘api_response.csv’ (https://github.com/Sunbio1/DataXflowGen).

To generate a larger model directly based only on the genes of interest, the first prompt for a one-shot model (Fig. S8; sup) can be used. The difference from the initial model generation pipeline is the starting prompt (Fig. S6, sup). In our case, we used the genes (ATM, CDK2, CDK4, CDKN1A, MAPK14, MTOR, NR1H4, PCNA, RB1, SPI1, TP53, and ZFP36L1) from the result of the last step of the original model (Fig. 3). The subsequent steps from the initial model pipeline are simply executed after the prompt (Fig. S7). If genes are to be added to the list, you can simply use the original pipeline and specify the number of genes to be added.

It is possible to change the prompt to obtain the best result for different analyses, e.g., for a cancer model.

### New gene list and list of gene pairs

Next, we convert the GenAI output into a format that can be systematically queried for interactions. To do this, we clean up the gene list, standardize the gene names, and generate all relevant gene pairs so that every possible regulator-target combination can be analyzed in a structured and reproducible manner.

This output was then processed (‘1_prompt.py’ (available here https://github.com/Sunbio1/DataXflowGen); python (version 3.13.1)). This included filtering out the gene list from the response and standardizing gene names, when necessary, e.g., TGF-ß to TGFb can be replaced by ‘str.replace()’ method in Python (can be extended in script: 1_prompt.py). The gene list served to generate gene pairings to analyze the interaction between all the genes and construct the interaction network. This list of gene pairs is structured as gene1, gene2; gene2, gene1; gene1, gene1; and gene2, gene2. In this context, gene1 influences gene2, and so on. Output file ‘extended_gene_pairs.csv’ can be found here (https://github.com/Sunbio1/DataXflowGen). These pairs may subsequently be used in the iterative single interaction question prompt. This single query is intended to reduce the hallucinations of GenAI. The newly generated gene list will be used to normalize the scRNA data for DataXflowGen and can be found above in the “Methods” section under Data-specific normalization.

### GenAI response for gene interaction

In this step, we convert GenAI’s text-based knowledge into a machine-readable description of which gene regulates which in the network and with what sign. By querying GenAI for one gene pair at a time and forcing it to select a single interaction type (activation, inhibition, indirect interaction, or no interaction), we reduce hallucinations and obtain a clean, standardized interaction table that can be used for subsequent modeling.

For this, an iterative loop was executed to query the interactions for each gene pair via GenAI to overcome hallucinations. Analyses were implemented in Python 3.13.1 using the standard library (including itertools) (‘1_2_prompt.py’).

The output of the interactions per gene pair was defined in the prompt (Fig. S8) as follows. Direct activation should be indicated with 1 or direct inhibition with − 1. In addition, indirect connections were also queried, which were to be defined as 2 for indirect activation or − 2 for indirect inhibition. Indirect interactions are interactions between genes where genes that convey the interaction are not present in the model, but via such genes, there is an interaction between the corresponding genes. When no information was available, this should be noted as 0. Therefore, information on the interactions of each gene pair can be displayed using these classifications. Based on the highest probability, the GenAI was only permitted to provide one response per gene pair. The output was filtered (Python version 3.13.1) and named ‘cleaned_gene_interactions.csv’ (available here https://github.com/Sunbio1/DataXflowGen).

### Generation of an adjacency matrix

We then convert the list of pairwise interactions into a mathematical object that can be processed using graph- and ODE-based modeling tools. The resulting weighted adjacency matrix records, for each ordered gene pair, whether a regulatory connection exists and its sign, provides a compact representation of the network structure.

This weighted adjacency matrix was generated based on the GenAI response (‘2_ad_matrix.py’). In the matrix A, each gene (node) is mapped to an index (node_to_index), and an N × N matrix is initialized with zeros. For each interaction, the indices u and v are derived from Gene1 and Gene2, respectively, and the interaction value is recorded as the edge weight at A[u,v]. This results in a directed graph as only the edge Gene1 → Gene2 is represented, while non-existent edges stay zero. The matrix data is sourced from the file 'cleaned_gene_interactions.csv’ and has exactly three columns: gene1, gene2, and interaction. The gene names are collected and arranged in alphabetical order, from which a node list is constructed to form the adjacency matrix A (‘adjacency_matrix.csv’).

### Updated adjacency matrix

To avoid redundant and unnecessarily complex models, we simplify the adjacency matrix by removing indirect edges that are already accounted for by paths of direct interactions. This pruning step can reduce the number of free parameters in the resulting ODE model, while preserving the network’s essential causal structure.

To this end, the extended adjacency matrix (extended_adjacency_matrix.csv) was processed to eliminate redundant indirect connections (4_update_matrix.py). This is optional, but it can reduce free parameters in the model since regulation via indirect interaction is canceled if a corresponding path of direct interactions exists. Two redundancy rules were implemented:

1. Positive redundancy: Any indirect activation (weight = 2) from gene i → j was removed if j was reachable from i through a path consisting solely of direct activations (edges with weight = 1).
2. Negative redundancy: Any indirect inhibition (weight =  − 2) from gene i → j was removed if j was reachable from i exclusively via direct inhibitory edges (weight =  − 1).

The existence of paths was checked using breadth-first search in the graph filtered by the corresponding edge. Updates were implemented solely when the redundancy criteria were satisfied. The cleaned matrix was then exported (4_update_matrix.py) both in the original CSV layout, including headers (modified_adjacency_matrix.csv), and as a pure numerical matrix (updated_adjacency_matrix.csv).

### Topology: create file for yED

For visualization and manual review, we export the final network into a format that can be opened directly in the yED Graph Editor. This step converts the adjacency matrix into a human-readable network diagram, in which nodes represent genes and edges indicate activating or inhibitory interactions.

In this step, the final adjacency matrix (modified_adjacency_matrix.csv) was converted so that it can be read by the yED software. This involved converting the matrix into an edge list containing only non-zero entries as triplets (Gene1, interaction, Gene2). The interaction signs were then converted to sign notation: “+” for 1/2 (activation) and “−” for − 1/ − 2 (inhibition) (‘yED.py’).

At the same time, a sorted list of unique genes representing the union of Gene1 and Gene2 was created. The output table was structured so that the interaction triplets occupied the first three columns, followed by an empty column and a fifth column listing all the unique genes. The table was padded to align both lists by row, then exported without headers as 'topo.xlsx’ (’yED.py’).

This file can be opened with yED software (https://www.yworks.com/) and converted to a readable Graph Markup Language file (‘graphml.file’). Briefly described in Crouch et al.^23^.

### Model fitting by D2D

In this step, we verify whether the proposed GRN can indeed explain the experimental data. To this end, the network is converted into a system of differential equations, and the parameters are estimated so that the simulated gene expression dynamics match the measured values as closely as possible. Concurrently, Data2Dynamics (D2D) maintains the model as simple and robust as possible. We then systematically examine which regulatory interactions are actually necessary and which can be removed without compromising the model’s explanatory power. To accomplish this objective, we implement L1 regularization to reduce the influence of non-sensitive parameters to zero and utilize an automated intervention analysis, termed ‘Lever’ (suggestions for additional starting points in the model), to investigate the impact of alterations in individual parameters on specific genes. This approach facilitates the identification of the most influential levers within the network.

For this, the interaction network (‘new_model.def’) is transferred to model equations so that the corresponding model can be fitted to the experimental data (‘new_data.def’ and ‘new_data.csv’) via the D2D framework^23,30^ (Fig. 5) and used to test whether the interaction network cannot be rejected given the data. After compilation, the parameter limits were cleaned up before optimization to avoid implausible parameter deviations (e.g., by restricting h_* to [1,10], b_6_2 to [0,5], and limiting the upper bounds of a_* to ≤ 50 while enforcing valid boundary intervals included as sanity bounds before fit starts (‘setup.m’). An initial fit was conducted and saved in PEtab format (‘export_full’). Furthermore, for each gene per time point, Chi-2 values have been stored in CSV format by using experimental data as the expected outcome and the corresponding values of the variables of the model (observables) as the observed outcome, normalized by their respective experimental (expected) standard error (‘chi2_full.csv’).

L1 regularization was used as a sensitivity test for the stability/redundancy check. After compilation, the parameter limits were cleaned up before optimization to avoid implausible parameter deviations (e.g., by restricting h_* to [1,10], b_6_2 to [0,5], and limiting the upper bounds of a_* to ≤ 50 while enforcing valid boundary intervals).

Subsequently, using qL1reg, an L1 penalty of 50 was applied to the subset of interaction parameters with the prefixes a_, b_, h_, delta_. The purpose of this regularization was to identify parameters with little influence and to reduce model complexity. Parameters classified as negligible by L1 fitting were removed from the set of free parameters during the subsequent optimization (qFit = 0), thereby simplifying the model structure. The reduced model was then refitted without L1 regularization, using the L1 solution as an initial estimate to obtain unbiased goodness-of-fit and model selection metrics. These were summarized by Chi-2, degrees of freedom (ndata − k, where k represents the number of fitted parameters), and information criteria (AIC and BIC), which were calculated from Chi-2 and k. Parameters with an absolute value below a predefined threshold (|p|< 10) were set to their L1-estimated values rather than being explicitly set to zero. Accordingly, the L1 method served primarily to identify the parameters with the greatest influence on the model. The reduced fit was exported in PEtab format, and the retained interactions were saved in CSV. In summary, after the L1-regularized fit, any parameters with absolute values below the predefined threshold (|*p*|< 10) are fixed and removed from the set of free parameters. The model is then refitted without regularization using the reduced parameter set. If the Chi-2 value obtained from the (unreduced) fit (‘chi2_full.csv’) and the Chi-2 value obtained after refitting (‘chi2_reduced.csv’) of the reduced model are essentially identical, the fixed parameters are considered redundant, and the fit is considered stable in terms of parameter reduction.

Next, we performed an automated intervention analysis, which we refer to as ‘Lever’ (implemented in ‘setup_all.m’), to systematically identify influential model parameters (‘levers’) whose perturbation most improves the fit of selected target genes. It provides an overview of whether each gene needs to be upregulated or downregulated in the model to improve the fit. The gene regulation file (all_genes_with_regulation.csv) explicitly defined the target nodes (genes) and the desired direction of change (up- or downregulation). For each such target with a high contribution to the overall Chi‑2, the model candidate parameters associated with that target were systematically adjusted (rather than the gene expression values themselves; increased or decreased by specific factors), and the model was re-simulated. The parameters were then evaluated based on the resulting change in the target response (e.g., change in peak value and/or area under the curve) and on how much they reduced the Chi‑2 contribution of these poorly fitted genes, thereby creating a prioritized list of lever parameters. We always focus on the genes with the highest Chi-2 values, as these are the points where the model explains the data the least well. By improving the fit for these genes, we reduce most of the discrepancy between the model and the experimental data. The results are stored in a timestamped diagnostic folder.

Model fitting was performed on a Linux x86_64 server (kernel 6.10.5–1-default) in MATLAB R2023a (9.14.0.2206163) using ‘*lsqnonlin’* (Optimization Toolbox v9.5), and automated intervention analysis (‘Lever’) was carried out with custom MATLAB utilities (e.g., ‘auto_from_csv’, ‘auto_find_levers’) integrated into the D2D workflow.

### Visualization

To make the fit of the model immediately apparent, we visualize how well the model fits the expression time curves of the individual genes. These diagnostic plots compare experimental data with simulations side by side, highlighting where the model fits well and where it deviates, which helps us identify parts of the network that may need to be refined.

For this purpose, a Python post-processing script (’matlab_plots.py’) was employed to visualize the final Chi-2 output (‘chi2_final.csv’, ‘chi2_full.csv’) and to compare the experimental data with the fitted model simulations. The CSV file was imported, and all distinct observation variables were presented in a multi-panel format (three columns; the number of rows dictated by the quantity of observation variables). For each observational variable, the experimental data were represented as points with error bars (± 1 effective standard error, Std) at their respective time points (Time), while the associated model predictions (SimulatedData) were plotted as a continuous curve. The total Chi-2 contribution of each observation variable (the sum of Chi-2 across all time points) was presented in the title of the sub-diagram (e.g., “χ^2^ …”). The corresponding Chi-2 value represents a measure of the goodness of fit of the corresponding gene and can be used as a pointer where the model fits the data worst and therefore needs improvements most. The axis formatting was constant throughout all panels, including a fixed x-range, specified tick spacing, consistent font size, y-ticks ranging from 0 to 1 in intervals of 0.1, and grid lines. The figures were exported in PDF and high-resolution raster formats (PNG/JPEG/TIFF; 600 dpi) for publication-quality reports.

### Back-loop preparation

In the back-loop phase, we prepare the model for a targeted feedback loop with GenAI by specifically identifying the points where the current network cannot reproduce the data. We quantify the deviation for each gene, identify which genes are explained the least well, and use this to determine whether they should be more strongly activated or inhibited in the model. This information then serves as structured input for the subsequent GenAI-guided expansion of the network.

### Data processing and Chi-2 evaluation

In the next steps, we establish a robust feedback loop between the data and the model. First, we quantify how well each gene is currently fitted so that we can focus the subsequent GenAI-guided refinements precisely on the parts of the network that explain the data least effectively.

To do this, the downstream evaluation of model performance (the file ‘chi2_full.csv’ (normal fit results) or ‘chi2_reduced.csv’ (fit results after model reduction)) is processed using ‘1_high_chi2_gen.py’. The misfit per variable (gene) was calculated by aggregating all entries for each variable name and summing the associated Chi-2 contributions across all time points. To maintain uniformity in nomenclature across all data sources, the suffix ‘_obs’ was eliminated from all variable names.

The table containing the total Chi-2 values for each variable was exported as ‘chi2_sums_per_variable_full.csv’. The variable exhibiting the biggest difference between simulation and experiment was identified by sorting the Chi-2 table in descending order, extracting the variable with the highest total Chi-2 value, and saving it as ‘full_gene_with_highest_chi2.csv’. The rationale behind this sorting is that we need to improve the model with the highest Chi-2 values because Chi-2 values are a measure of misfit, and genes with the highest Chi-2 contribute most to the discrepancy.

A filtered table was created from either ‘chi2_full.csv’ or ‘chi2_reduced.csv’, which only contains variable names, experimental values (ExpData), simulated values (SimulatedData; model output of the corresponding gene), and data index (DataIndex). The suffix ‘_obs’ was removed from the variable names, and the resulting dataset was exported as ‘f_filtered_data.csv’ to enable a direct comparison between experimental measurements and simulated outcomes.

### Post-processing and regulation assignment

Next, we determine in which direction the model needs to be adjusted for the gene with the poorest fit. By comparing the simulated and experimental values, we conclude whether this gene should be more strongly activated or inhibited in the model to improve the fit and encode this as a simple regulatory label (activation or inhibition) for the next GenAI step.

To determine whether the gene with the highest Chi-2 value needs to be more strongly activated or inhibited to improve the agreement between the model and experimental data, we applied the post-processing script ‘2_activation_inhibition.py’. The filtered simulation-experiment table (‘f_filtered_data.csv’) and the file containing the variable with the highest Chi-2 value (‘full_gene_with_highest_chi2.csv’) were imported into this script. It then identifies the corresponding gene and extracts all matching entries from the main dataset. The script then calculates the mean of the simulated and experimental values for this gene and determines their ratio. If the simulated values exceed the experimental values (ratio > 1), the deviation is interpreted as an overestimation, indicating inhibitory regulation. Conversely, the deviation is interpreted as an underestimation if the simulated values are lower than the experimental values (ratio ≤ 1), which corresponds to activating regulation. The derived regulation type is finally appended to the gene-specific output file.

The variable name associated with the highest Chi-2 value (in total) was obtained from the initial row of ‘full_gene_with_highest_chi2.csv’. Here, the gene-specific Chi-2 values were calculated as the sum of the Chi-2 contributions across all time points for each gene. The corresponding time-resolved experimental and simulated values for this variable were taken from the file ‘f_filtered_data.csv’. The ratios of the simulated values (SimulatedData) and the experimental values (ExpData) were obtained for this variable. The ratio of the simulated mean to the experimental mean indicated the simulation bias.

Variables with ratios beyond one (indicating simulation values are bigger than experiment values on average) are referred to as inhibition, whereas ratios less than or equal to one were classified as activation. A new column (Regulation) for the assigned regulation category was incorporated into the table of top variables, which was then exported as ‘gene_with_regulation.csv’.

### Iterative back-loop via GenAI

These steps close the loop between the data, GenAI, and the network model. Starting with the gene that has the poorest fit and the required direction of change, we use GenAI to propose new upstream regulators, integrate them into the network, and re-evaluate the model, thereby expanding the network in a targeted, data-driven way.

### Candidate target identification

In this step, we use the current model fit to decide which gene to improve next and in which direction. We select the gene with the highest misfit value (largest Chi-2 value) and the corresponding regulation (activation or inhibition) and treat it as the target for refinement. This provides a targeted starting point for the subsequent GenAI query, which then searches for plausible upstream regulators of precisely this gene to improve its fit.

For this, we integrate a new gene (node) into the network, as it could significantly influence the system dynamics (‘1_back_loop.py’). For this purpose, the candidate target gene with the highest Chi-2 value and the required interaction regulation (activation or inhibition) was loaded from ‘gene_with_regulation.csv’. Additionally, the existing genes from the network were loaded from ‘loop_gene_list.csv’. This ensures that these genes are not included in the GenAI query again in order to obtain new genes for model development. To propose an upstream regulator for this target, the regulatory direction (activation or inhibition) was first determined using the post-processing script ‘2_activation_inhibition.py’. This information was then incorporated into a constrained prompt (Fig. S9, Supplementary Material), which was queried deterministically (temperature = 0, top_*p* = 0) to enforce a strict upstream causal relationship (‘2_activation_inhibition.py’). This excluded downstream targets, co-factors, and non-causal interactors, prioritizing regulators prevalent in fibroblast senescence models that can typically be detected in bulk or single-cell RNA-seq datasets. Regulators that were already present in the current network were excluded (‘loop_gene_list.csv’). The prompt instructed the model to output either one HGNC gene symbol or ‘EMPTY’ if no suitable candidate met all the criteria from the literature and database research. The GenAI response was saved as ‘back_loop_api_response-csv’. Problems can arise if the regulatory genes suggested by the AI have only a very minor influence on the target node. In such cases, one can specifically ask the AI to identify, for example, five candidate genes that are expected to regulate the respective node. Subsequently, the normalized file or table can be used to assess how strong the expected regulatory contribution of these genes is. Determining which of these candidates are ultimately included as nodes in the model typically requires additional expert knowledge and a manual comparison with the biological context. Another option is to directly provide the GenAI with a count list, DEG list, or similar input data so that it can identify suitable genes based on this information. This approach can facilitate the selection of biologically relevant candidates but typically requires a large number of queries and thus higher token consumption, which in turn entails correspondingly higher costs. It should be noted that only genes that were also present in the dataset were used to expand our model. It is also possible to use a prompt (Fig. S10; sup) that is not as strict as the one in Fig. S9 in the supplementary. This allows genes to be identified when the strict model only outputs EMPTY because the information is not yet sufficient. It is also possible to include other factors, e.g., not only in fibroblasts, or also from the oncolytic field, as the available information on gene interactions in these areas is usually more extensive. The normalization step (see methods: Data-specific normalization) was used to check whether the gene was present in the dataset. It is important to note that if the gene was not present in the dataset, the name assigned by GenAI may be a synonym. This was verified using the ‘GeneCards’ database^109^, a database of human genes, and the name was replaced if necessary. It is also possible to make the entire DEG list available to GenAI and use it to identify a suitable gene, but this can be costly. If none of the genes suggested by GenAI are present in the dataset, the model structure offers another option (‘subgraph.py’; ‘upstream_root_cause.py’). Based on it, regulators already anchored in the network can be identified that could potentially exert a stronger influence if a new regulatory node is made available to them. Furthermore, the model allows for the inclusion of hypothetical nodes (genes) for which no experimental data are available. Such nodes can be incorporated into the model as constant nodes, thereby representing their presence and regulatory influence without requiring explicit time-series measurements. This strategy can also be used to preliminarily assess the potential effects of such a hypothetical node on the network. Furthermore, it may reveal that a regulatory interaction not yet described is required in this part of the network, which could prompt further experimental or literature-based investigations.

### Gene-pairs generation for back loop

We then integrate the new regulator into the existing network by identifying all relevant gene pairs that should be examined for interactions. This ensures that every possible connection between the new gene and the existing network is systematically evaluated, rather than simply being added on an ad hoc basis.

To do this, the upstream regulator was imported from the file ‘back_loop_api_response.csv’ (‘2_create_genepairs.py’). Formatting artifacts from the code block, such as triple backticks and optional language tags, as well as empty lines, were removed to yield a single valid HGNC symbol. The network gene list was loaded from ‘loop_gene_list.csv’, and directed gene pairs were formed between the predicted regulator (gene that is supposed to improve the model fit by its regulation) and each network gene. For each combination, both orientations (regulator → gene and gene → regulator) were generated, excluding self-pairs, to embed the new gene into the existing regulatory network. Finally, the self-interaction (regulator → regulator) was added. All resulting pairs were compiled into ‘gene_pairs.csv’ with columns GeneA and GeneB. Next, we need to check if the added gene improves the model fit.

### LLM-based GenAI interaction for searching for target genes

As before, we query GenAI in pairs to determine which of these potential connections are supported by the literature and what their sign is (activation, inhibition, or no interaction). This step converts the potential connections of the new regulator into a machine-readable set of interactions that can be merged with the existing network and validated against the data.

For this purpose, all directed gene pairs for the added gene from gene_pairs.csv were queried individually using deterministic model inference (model = ‘Sonar’, temperature = 0, top_*p* = 0). For each pair (gene1, gene2), the model was instructed to return a single integer encoding the direction and type of influence related to fibroblast senescence. The following notation should be used: − 1 = direct inhibition, − 2 = indirect inhibition, 1 = direct activation, 2 = indirect activation, 0 = no known interaction or insufficient evidence. The prompt required evidence from multiple peer-reviewed publications and prohibited speculative associations. If there was no repeated literature support, the model was restricted to return 0. The output was saved in ‘gene_interactions.csv’. The interaction values from previous runs are left unchanged.

In a further cleaning step, the “Interaction” field was analyzed using regular expressions to obtain the first signed integer. Before the analysis, all Markdown formatting, brackets, and line breaks were removed. The file ‘cleaned_gene_interactions.csv’ was created from the standardized results (‘3_back_loop2.py’).

### Adjacency-matrix extension

We then merge the newly derived interactions with the existing network and update the adjacency matrix so that the expanded model can be simulated and analyzed in the same way as before.

For this purpose, the base adjacency matrix (‘loop_modified_adjacency_matrix.csv’), with the gene symbols serving as the row and column indices, and the cleaned interaction table (‘cleaned_gene_interactions.csv’) were loaded. All genes appearing in either the Gene1 or Gene2 fields were combined with the genes in the matrix of the previous regulatory network (baseline) to create a unified, sorted list.

To create a unified adjacency matrix, a new square matrix was initialized with zeros. Entries from the baseline matrix were copied, and each new interaction was inserted by assigning the specified interaction value to the corresponding position (Gene1 or Gene2). The extended matrix was written to the file ‘extended_adjacency_matrix.csv’ (‘4_extended_ad_matrix.py’).

### Update the adjacency matrix (loop)

As with the initial model, unnecessary indirect edges are removed to ensure that the network, which has been expanded to include loops, is as simple and easy to understand as possible.

For the loop-extended model, the adjacency matrix must be updated using the procedure described in the “Initial model generation pipeline” section (updated adjacency matrix). Briefly, indirect edges were removed if an equivalent directed path composed solely of direct edges of the corresponding weight existed (BFS-based path check: ‘5_loop_up_matrix.py).

The resulting loop-specific outputs are provided as ‘loop_updated_adjacency_matrix.csv’ and ‘loop_modified_adjacency_matrix.csv’.

### Create a file for yED (loop)

Finally, we export the updated loop network to yED to visually inspect how the newly added regulator integrates into the existing structure.

For this, the loop network was exported in a yED-compatible topology format. The file contains the following: (i) the regulator gene (column 1), (ii) the interaction sign, either “+” (activation) or “−” (inhibition) (column 2), (iii) the target gene (column 3), (iv) an empty spacer column (column 4), and (v) a full list of all genes (column 5). The loop topology table is provided as loop_topo.xlsx and can be opened in yED^110^ and exported to GraphML, as described in Crouch et al.^23^.

### Evaluation of GenAI response

Two questions were asked to evaluate the response of GenAI. One question was how the GenAI model’s performance compared to random models. To answer this question, ten random models with specific settings were generated. Based on the iteratively generated GenAI model from Fig. 3, these included all nodes, as well as the number of edges and self-loops, having to remain the same. If a node had no incoming edges, the random model should also contain the same number of nodes with no incoming edges. This ensures that the free parameters remain the same for all models. Each model was adjusted and checked for goodness-of-fit based on Chi-2 value, *p*_value, BIC, AIC, and AICc (see Table 1). How this test was obtained is shown in the “Methods” section in the supplementary material.

The second question was how GenAI behaves deterministically when three additional genes (nodes) are added to an existing network (Table 2). This was merely intended to show whether GenAI remains sufficiently deterministic in its responses based on the prompt used and the settings applied. To this end, GenAI was asked to name three genes that might be suitable for the existing model (Fig. 3). The results demonstrated that the method is sufficiently deterministic, as the same three genes were consistently identified as potential regulators for the existing model in the queries. The selection of three genes was an arbitrary assumption without further methodological justification. The script explanation can be found in the supplementary material.

## Supplementary Information

Below is the link to the electronic supplementary material.

Supplementary Material 1

## Data Availability

This study reuses publicly available single-cell RNA-seq data from Wechter, N. et al., “Single-cell transcriptomic analysis uncovers diverse and dynamic senescent cell populations” (Aging, 2023, 10.18632/aging.204666). Specifically, we analyzed samples GSM7068361–GSM7068366 and GSM7068354 from the GEO Series GSE226225. All raw and processed data are available from the NCBI Gene Expression Omnibus under accession number GSE226225. No new datasets were generated in this study.
