# Supplementary material for: DataXflowGen for GenAI-driven model generation
Source: Sci Rep. 2026 Jun 6;16:17534. doi: 10.1038/s41598-026-56492-8 (PMC13242519; doi:10.1038/s41598-026-56492-8)
Supplement: Supplementary file 1 — Supplementary Material 1 [file 41598_2026_56492_MOESM1_ESM.docx]

**Table of Contents**

[Results 2](#_Toc230186017)

[Iterative GenAI modeling 2](#_Toc230186018)

[Methods 8](#_Toc230186019)

[Initial model generation pipeline – prompts 8](#_Toc230186020)

[Iterative back-loop via GenAI - prompt 10](#_Toc230186021)

[Evaluation 11](#_Toc230186022)

[Random model generation 11](#_Toc230186023)

[Batch fitting and evaluation procedure 12](#_Toc230186024)

[L1 regularization and re-fitting 13](#_Toc230186025)

[Additional Methods 13](#_Toc230186026)

[Upstream root-cause analysis and two-hop expansion 13](#_Toc230186027)

[Subgraph analysis of non-fitting genes 16](#_Toc230186028)

[List of Figures 17](#_Toc230186029)

[List of Tables 18](#_Toc230186030)

# Results

## Iterative GenAI modeling

Additional results from the subsequent steps of the iterative, GenAI-based modeling workflow are presented in the following supplementary figures (S1–S5) and in the attached summary table (Table SR1), which together provide detailed fit statistics and highlight the genes with the largest fit errors for each model configuration.
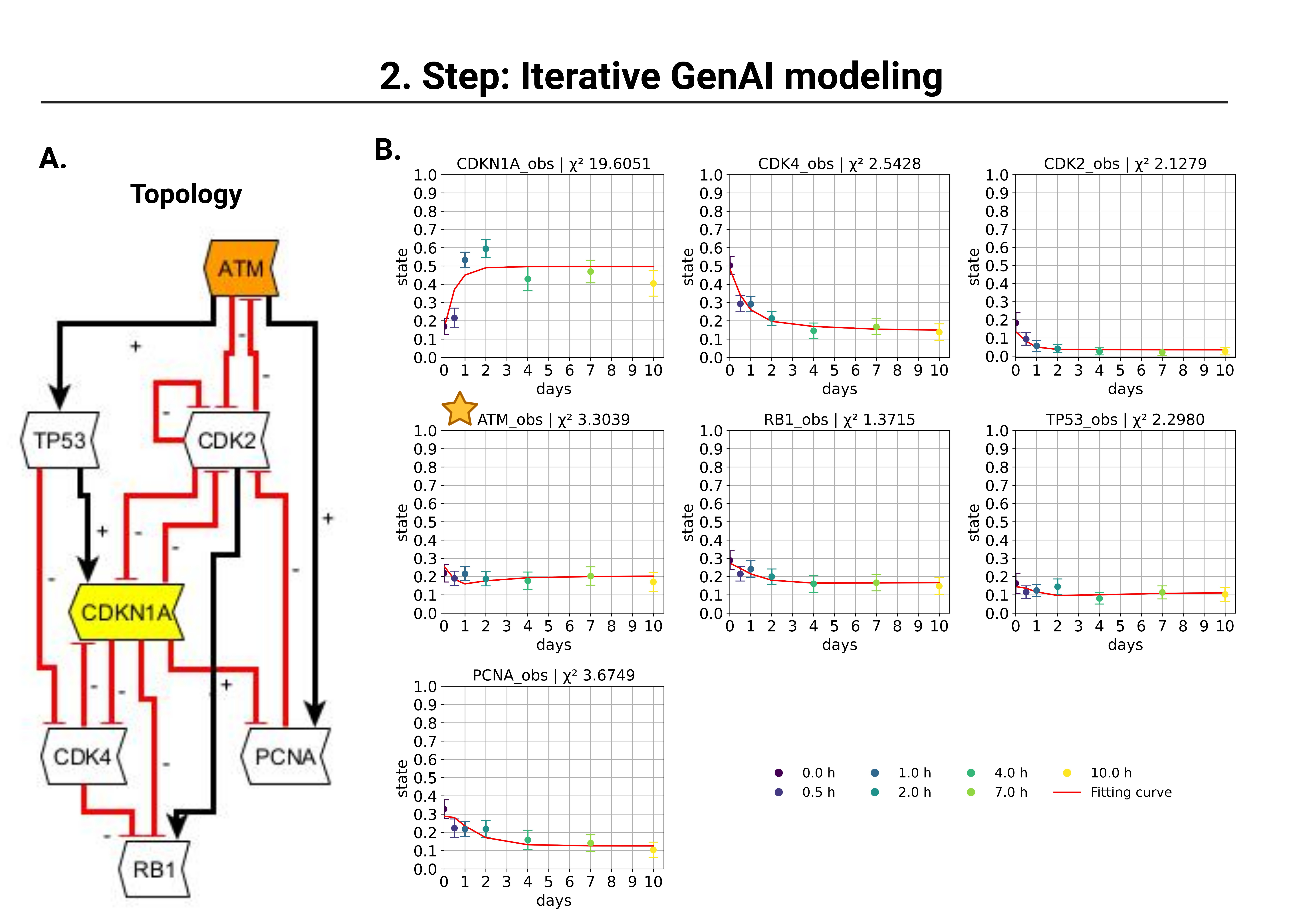


**Figure S 1: Second step of iterative GenAI modeling.** A: Topology of an interaction model with 7 regulatory nodes. Positive regulation is represented by black arrows, whereas negative regulation is indicated by red inhibitory arrows. The node outlined in yellow presents positive regulation by ETO treatment for the model. The orange node is new and has been integrated into the topology based on the GenAI response. B: Results of model fitting. Ten time points (days) are available for each gene with their representative state (between 0 and 1): 0 d (dark purple), 0.5 d (purple), 1 d (blue), 2 d (turquoise), 4 d (green), 7 d (light green), and 10 d (yellow). We provided Chi-2 values for each gene to evaluate the precision of the fit for the expression data. Total Chi-2 value equals 34.91 with 49 data points and 31 free parameters. Created in BioRender. Crouch, S. (2026) <https://BioRender.com/kesd6hk>


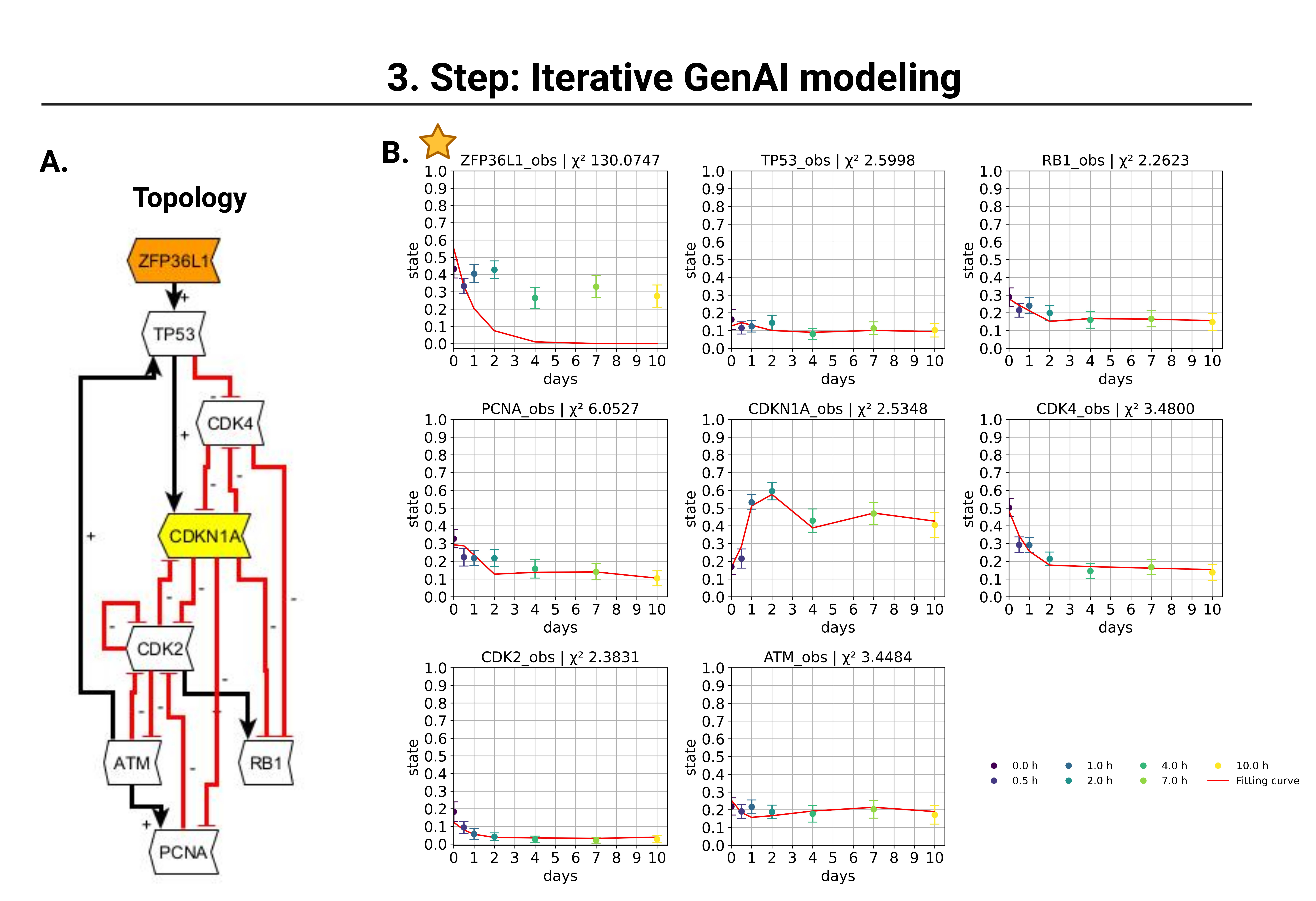


**Figure S 2: Third step of iterative GenAI modeling.** A: Topology of an interaction model with 8 regulatory nodes. Positive regulation is represented by black arrows, whereas negative regulation is indicated by red inhibitory arrows. The node outlined in yellow presents positive regulation by ETO treatment for the model. The orange node is new and has been integrated into the topology based on the GenAI response. B: Results of model fitting. Ten time points (days) are available for each gene with their representative state (between 0 and 1): 0 d (dark purple), 0.5 d (purple), 1 d (blue), 2 d (turquoise), 4 d (green), 7 d (light green), and 10 d (yellow). We provided Chi-2 values for each gene to evaluate the precision of the fit for the expression data. Total Chi-2 value equals 152.84 with 56 data points and 33 free parameters. Created in BioRender. Crouch, S. (2026) <https://BioRender.com/0wdvg46> .


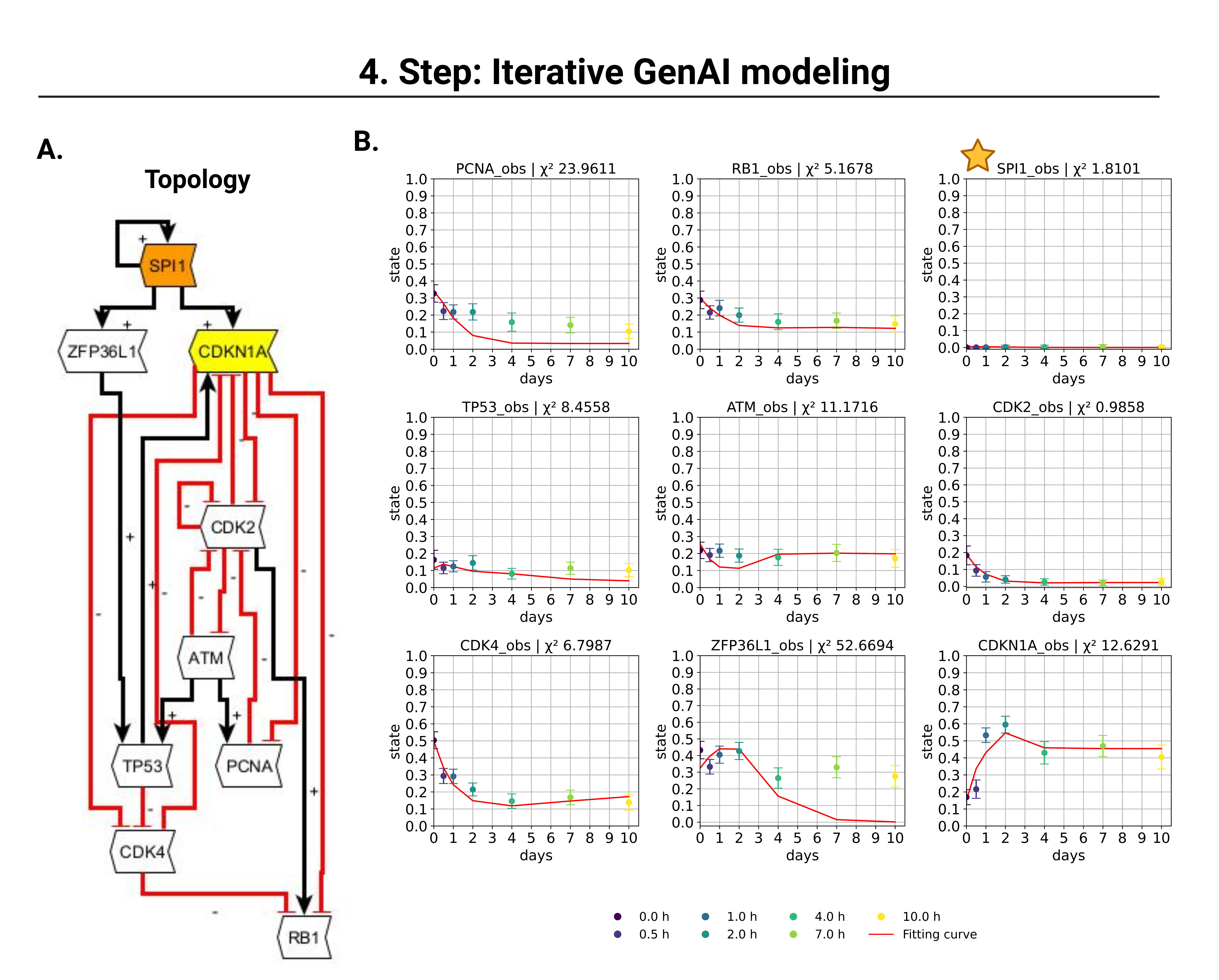


**Figure S 3: Fourth step of iterative GenAI modeling**. A: Topology of an interaction model with 9 regulatory nodes. Positive regulation is represented by black arrows, whereas negative regulation is indicated by red inhibitory arrows. The node outlined in yellow presents positive regulation by ETO treatment for the model. The orange node is new and has been integrated into the topology based on the GenAI response. B: Results of model fitting. Ten time points (days) are available for each gene with their representative state (between 0 and 1): 0 d (dark purple), 0.5 d (purple), 1 d (blue), 2 d (turquoise), 4 d (green), 7 d (light green), and 10 d (yellow). We provided Chi-2 values for each gene to evaluate the precision of the fit for the expression data. Total Chi-2 value equals 123.65 with 63 data points and 39 free parameters. Created in BioRender. Crouch, S. (2026) <https://BioRender.com/j1isrla>


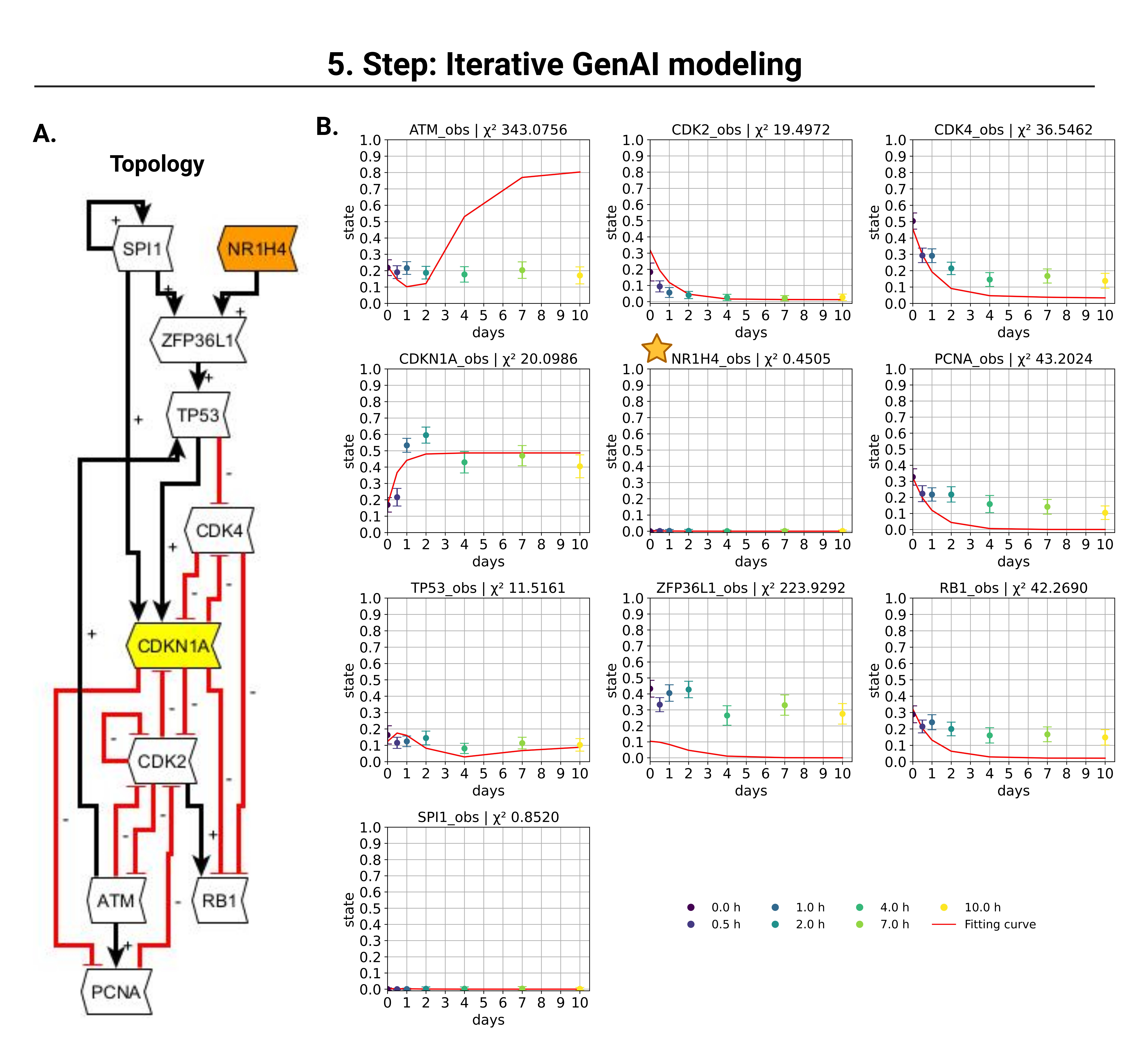


**Figure S 4: Fifth step of iterative GenAI modeling**. A: Topology of an interaction model with 7 regulatory nodes. Positive regulation is represented by black arrows, whereas negative regulation is indicated by red inhibitory arrows. The node outlined in yellow presents positive regulation by ETO treatment for the model. The orange node is new and has been integrated into the topology based on the GenAI response. B: Results of model fitting. Ten time points (days) are available for each gene with their representative state (between 0 and 1): 0 d (dark purple), 0.5 d (purple), 1 d (blue), 2 d (turquoise), 4 d (green), 7 d (light green), and 10 d (yellow). We provided Chi-2 values for each gene to evaluate the precision of the fit for the expression data. Total Chi-2 value equals 740.97 with 70 data points and 41 free parameters. Created in BioRender. Crouch, S. (2026) <https://BioRender.com/j3uffqj>


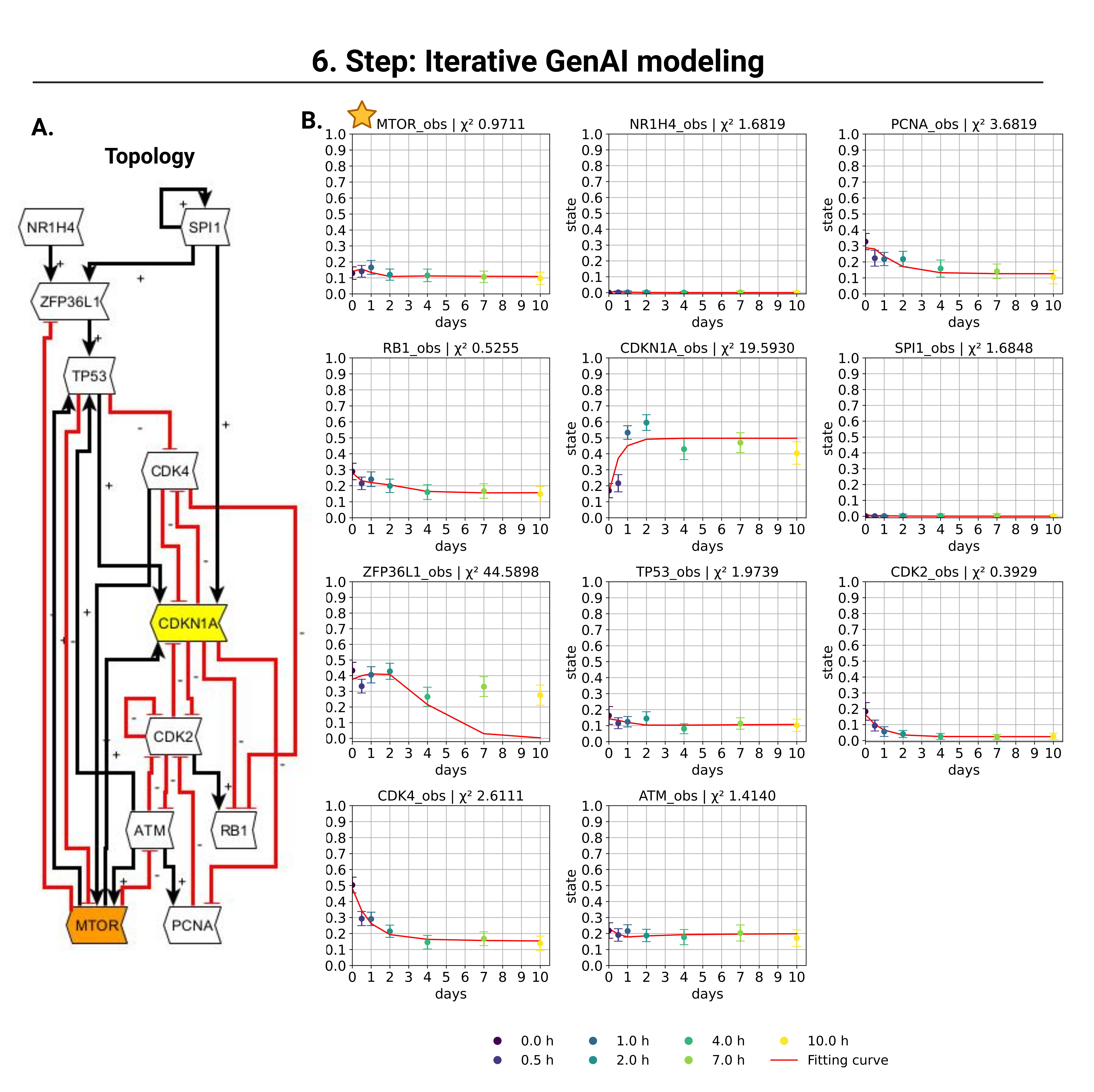


**Figure S 5: Sixth step of iterative GenAI modeling.** A: Topology of an interaction model with 7 regulatory nodes. Positive regulation is represented by black arrows, whereas negative regulation is indicated by red inhibitory arrows. The node outlined in yellow presents positive regulation by ETO treatment for the model. The orange node is new and has been integrated into the topology based on the GenAI response. B: Results of model fitting. Ten time points (days) are available for each gene with their representative state (between 0 and 1): 0 d (dark purple), 0.5 d (purple), 1 d (blue), 2 d (turquoise), 4 d (green), 7 d (light green), and 10 d (yellow). We provided Chi-2 values for each gene to evaluate the precision of the fit for the expression data. Total Chi-2 value equals 79.12 with 77 data points and 50 free parameters. Created in BioRender. Crouch, S. (2026) <https://BioRender.com/mpugtrd>

**Table SR 1: Fitting results of iterative modeling via GenAI response.** The table summarizes the goodness‑of‑fit and model compliance for the evaluated network configuration. For each fit, the Chi‑2 value, the number of data points, and the number of free parameters are reported, together with the corresponding p‑value and a binary decision on whether the model is compliant with the data at the 95% significance level. In addition, the table lists the newly introduced gene in the model and indicates which gene exhibits the highest per‑gene Chi‑2 contribution (here TP53 with Chi-2 = 33.87), highlighting the main target for subsequent root‑cause analysis and model refinement.

| **Fit** | **Chi-2** | **Data Points** | **Free Parameter** | **Model compliant?** | **New Gene** | **Highest Chi-2 Value** |
| --- | --- | --- | --- | --- | --- | --- |
| 1 | 60.77 | 42 | 24 | pval(dof=18) = 0.000002, model is NOT compliant with data for 95.00% sign. level  pval(dof=42) = 0.030431, model is NOT compliant with data for 95.00% sign. level | --- | TP53  33.87 |
| 2 | **34.91** | 49 | 31 | pval(dof=18) = 0.009708, model is NOT compliant with data for 95.00% sign. level  pval(dof=49) = 0.935691, model is compliant with data for 95.00% sign. level | ATM | CDKN1A  19.60 |
| 3 | **152.83** | 56 | 33 | pval(dof=23) = 0.000000, model is NOT compliant with data for 95.00% sign. level  pval(dof=56) = 0.000000, model is NOT compliant with data for 95.00% sign. level | ZFP26L1 | ZFP26L1  130.07 |
| 4 | **123.65** | 63 | 39 | pval(dof=24) = 0.000000, model is NOT compliant with data for 95.00% sign. level  pval(dof=63) = 0.000008, model is NOT compliant with data for 95.00% sign. level | SPI1 | ZFP36L1  52.67 |
| 5 | **740.97** | 70 | 41 | pval(dof=29) = 0.000000, model is NOT compliant with data for 95.00% sign. level  pval(dof=70) = 0.000000, model is NOT compliant with data for 95.00% sign. level | NR1H4 | ATM  343.08 |
| 6 | **79.12** | 77 | 50 | pval(dof=27) = 0.000001, model is NOT compliant with data for 95.00% sign. level  pval(dof=77) = 0.411808, model is compliant with data for 95.00% sign. level | MTOR | ZFP36L1  44.59 |
| 7 | **33.51** | 84 | 55 | pval(dof=29) = 0.257473, model is compliant with data for 95.00% sign. level  pval(dof=84) = 1.000000, model is compliant with data for 95.00% sign. level | MAPK14 | CDKN1A  19.61 |

# Methods

## Initial model generation pipeline – prompts

The subsection “Initial model generation pipeline – prompts” lists all prompts used in the GenAI workflow. The underlying method is described in detail in the manuscript.


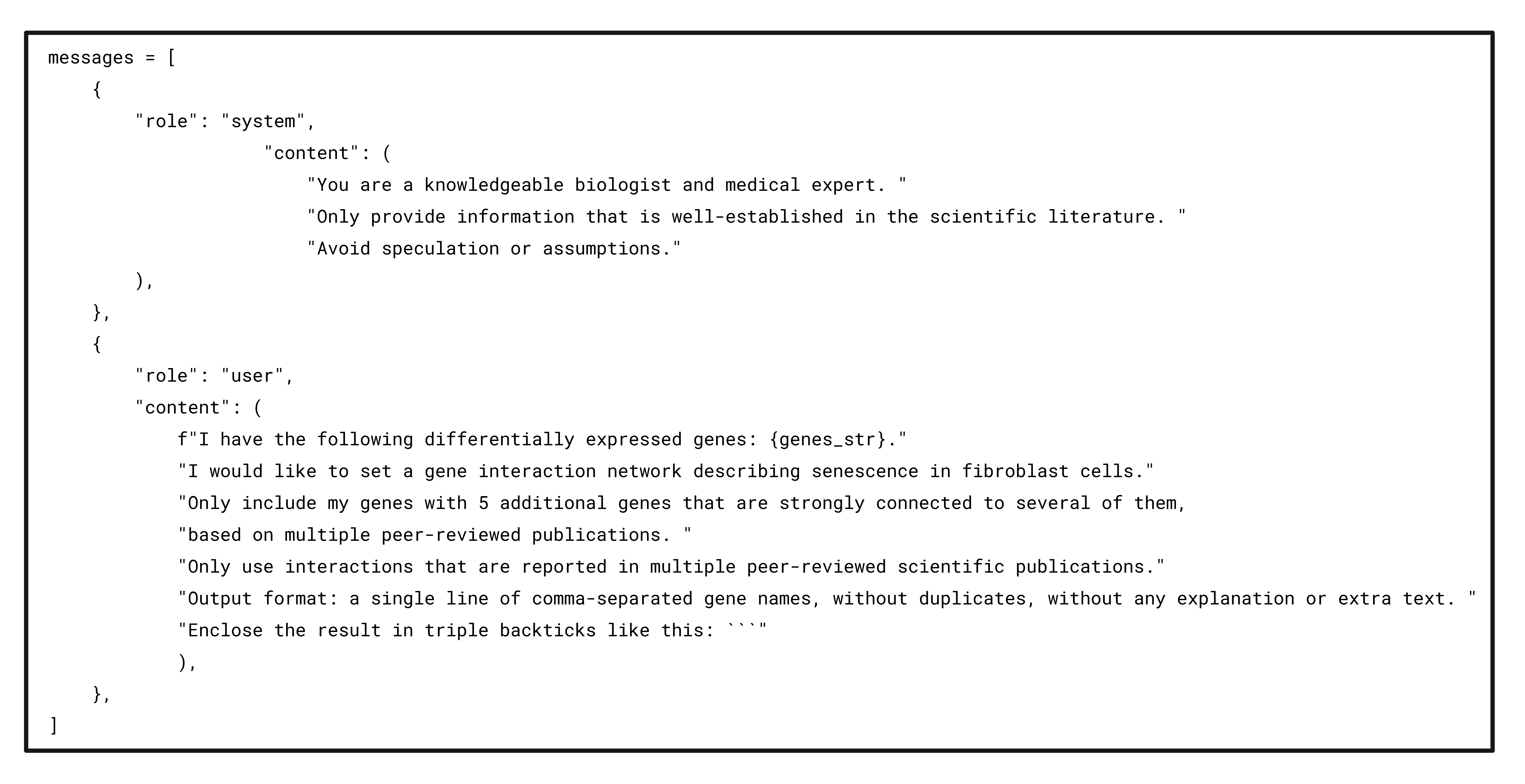


**Figure S 6:** **Prompt-based approach to generating a fibroblast-specific senescence gene interaction network.** The system defines the model as a biological and medical expert. The user provides a gene list and restricts interactions to those that have been published repeatedly. The system then enforces a standardized, comma-separated gene list as the model output. Created in BioRender. Crouch, S. (2026) <https://BioRender.com/348iuum>


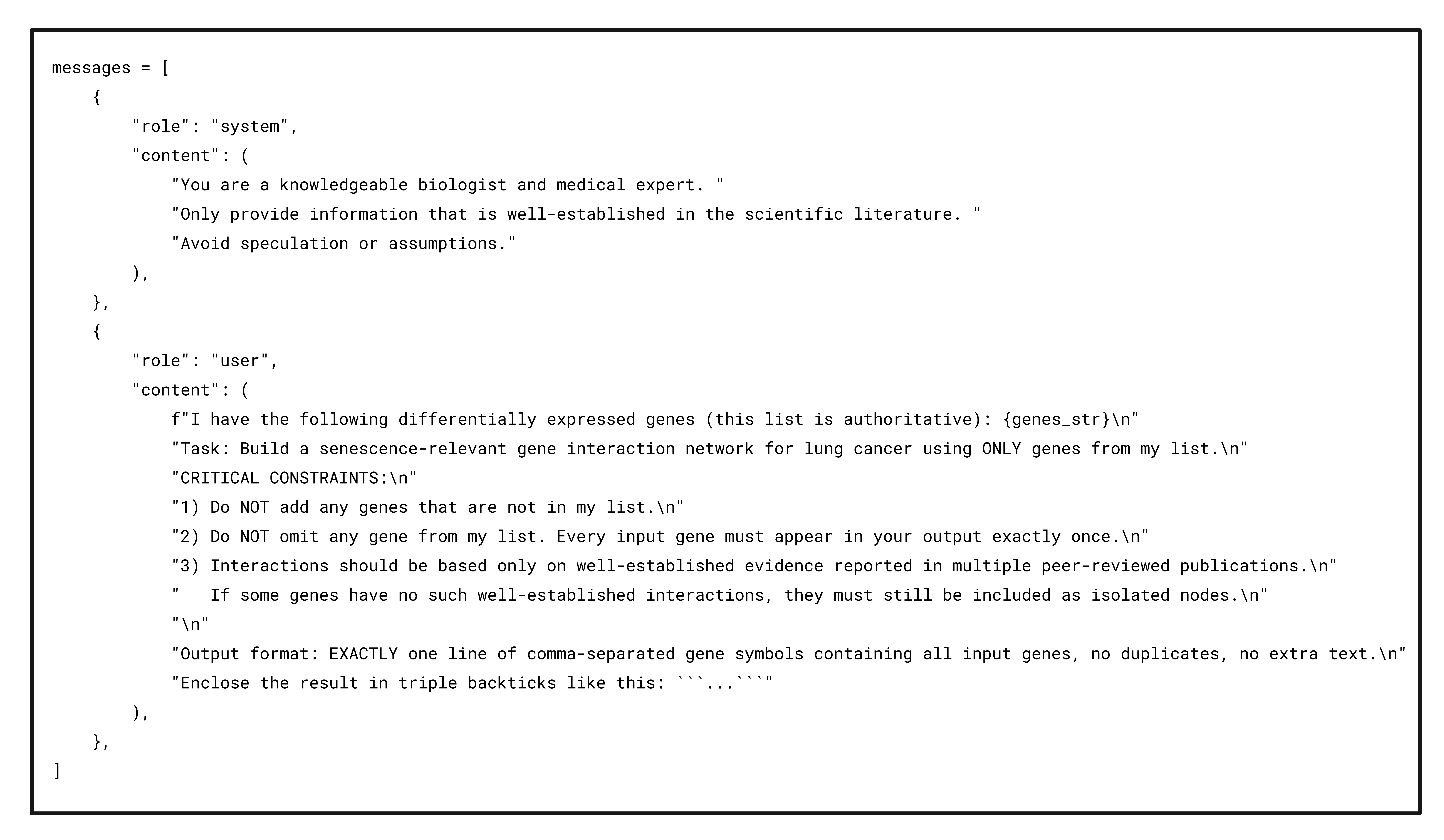


**Figure S 7****: One-shot model prompt for automated use of the analysis pipeline with genes of interest.** The system and user prompts define roles, inputs (relevant list of differentially expressed genes), senescence context (fibroblasts), and strict output formalities and are used solely to invoke LLM-based network construction in a reproducible manner without manual intervention. Created in BioRender. Crouch, S. (2026) <https://BioRender.com/63f53zs>


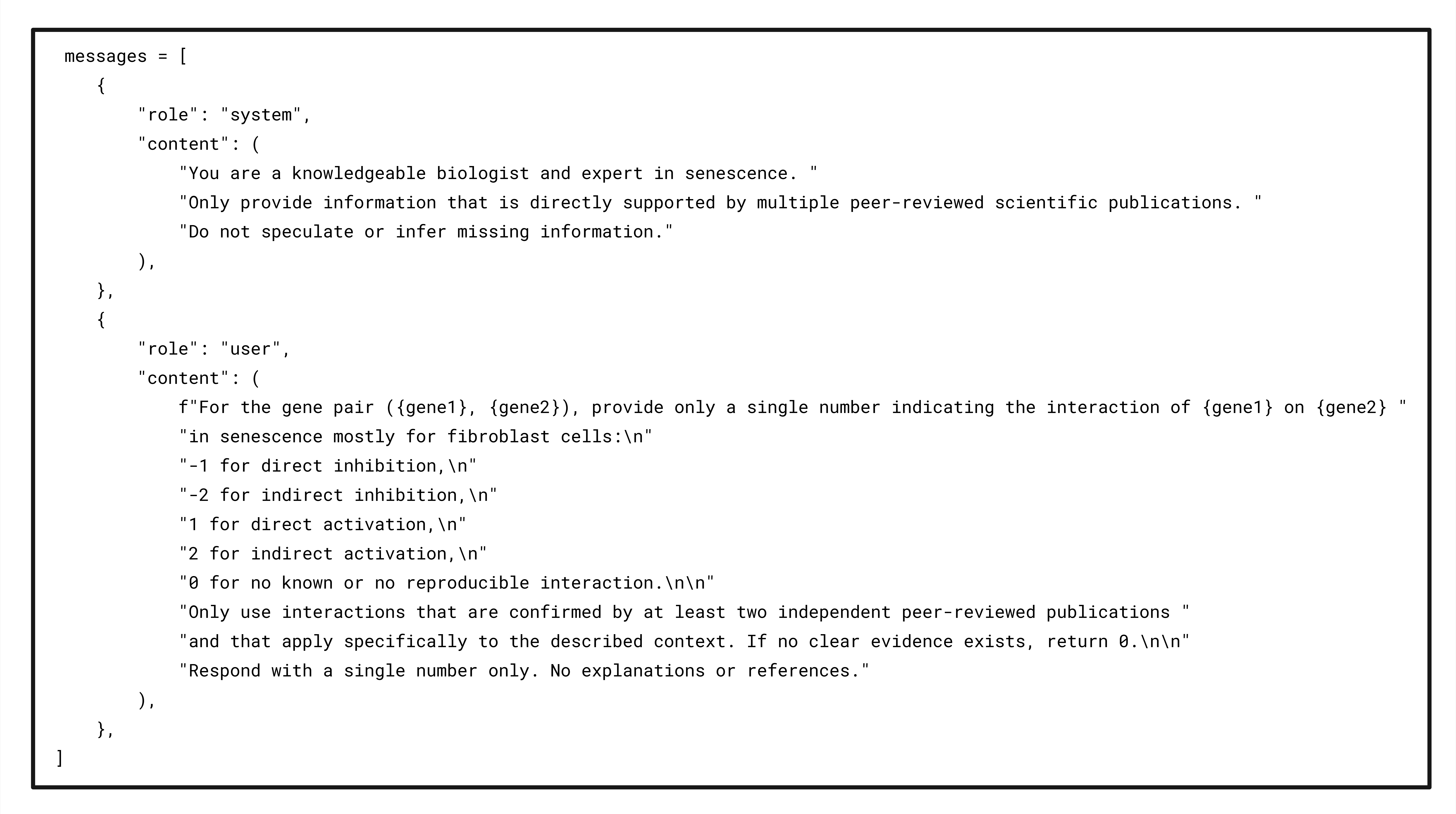


**Figure S 8****: Prompt for automated annotation of gene interactions in fibroblast senescence.** The model assigns each gene pair an integer (−2, −1, 0, 1, 2) indicating evidence-based activation, inhibition, or the absence of confirmed interaction. Created in BioRender. Crouch, S. (2026) <https://BioRender.com/jl7rcd6>

## Iterative back-loop via GenAI - prompt





**Figure S 9****: Back loop prompt for automated identification of upstream regulators for target genes.** The system and user messages enforce causal, direction-specific regulation (activation/inhibition), exclude genes already in use and fibroblast-distant, line-specific factors, and specify a strictly standardized output format (a single regulator symbol in backticks or ‘EMPTY’). Created in BioRender. Crouch, S. (2026) <https://BioRender.com/3z6feca>


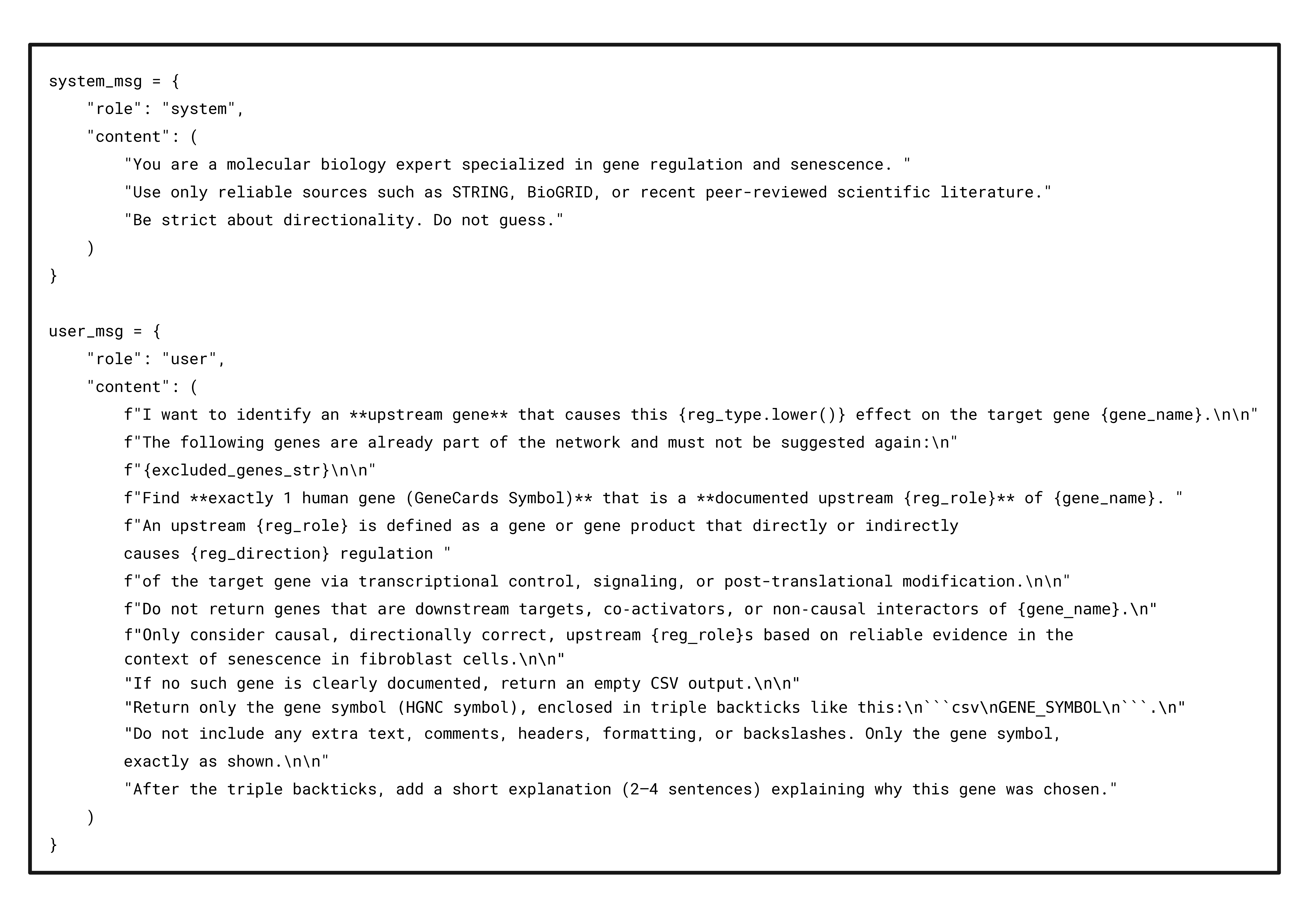


**Figure S 10****: Simple back loop prompt for identifying causal upstream regulators in senescence.** The system and user messages require a clearly documented, direction-specific regulator of target genes in fibroblasts from curated databases or current literature. Created in BioRender. Crouch, S. (2026) <https://BioRender.com/y20l4ha>

## Evaluation

### Random model generation

To test whether our GenAI-based model actually performs better than a random process, we generated a series of random networks. We started with the adjacency matrix of the final model and randomly redistributed its edges while preserving the total number of edges, their type (activation or inhibition), and the number of input-free nodes and self-regulating nodes. This resulted in random networks that are structurally similar to the GenAI model but contain only random structures and therefore serve as a basis for evaluating the added value of the network structure derived from GenAI.

Randomized adjacency matrices were produced from the adjacency matrix of the final step (Figure R 3) while adhering to stringent structural constraints (‘make_random_adjacencies.py’) and described in the following. Throughout shuffling, the global distribution of edge weights and the overall count of zeros were maintained. In each randomized matrix, exactly two “no-input” nodes also available in the GenAI model (Figure R 3) had to have no incoming edges (all off-diagonal entries in the corresponding column were set to zero), and two "self" nodes were required to be self-regulated (with a non-zero diagonal entry). One "overlap" node belonged to both categories (self-loop and no input nodes). This means that one of these two nodes had no input from other nodes and regulated itself through a self-loop. This was also set as strict for the random models. In addition, diagonal entries were restricted so that only self-regulated nodes could possess non-zero self-loops; all other diagonal entries were set to zero. Randomizations resulting in isolated nodes (lacking incoming and outgoing edges, excluding self-loops) were dismissed. Using various random seeds, 10 truly randomized matrices were produced and collected for subsequent study. These settings serve to make the models comparable by having the same free parameters for each model.

### Batch fitting and evaluation procedure

All GenAI-based and randomized networks were then automatically fitted to the data. For each model, we used two protocols: one without L1 regularization and one with L1 regularization followed by a re-fit, allowing us to compare both “full” and “thinned” models. Since we used the same optimization method, the same parameter bounds, and the same multi-start strategy for all models, the comparison of fit quality is fair and demonstrates how well the GenAI model performs relative to the random networks. This setup is intended solely to evaluate the quality of the GenAI-based model in relation to the corresponding randomized network structures.

All folders labeled r_* were processed through a batch pipeline and fitted according to two protocols: NO_L1 (uncontrolled) and L1_REFIT (L1-regulated structural fitting followed by unregulated refitting). For each run, the model and data configuration files were automatically identified and compiled in Data2Dynamics (D2D). All fitted parameters (qFit = 1) were initialized with a constant starting value of 0.2 and were restricted to specified parameter bounds. The lsqnonlin solver was used for optimization. In multi-start mode, an initial fit started from the fixed start, followed by a multi-start fit that employed Latin hypercube sampling (arFitLHS, nStarts = 50) and preserved the solution with the minimal data Chi-2 value.

### L1 regularization and re-fitting

We use L1 (Lasso) regularization to simplify the network in a targeted manner and keep only the most important regulatory parameters active. This makes the model more sparse, less prone to overfitting, and easier to understand from a biological perspective. At the same time, this step helps suppress parameters that are difficult to identify or numerically unstable and creates a more robust foundation for comparing different network structures.

L1 regularization promotes sparse parameterization by adding an L1 penalty term (L1penalty = 50) (equal to the sum of the absolute values of the specified parameters) to the objective function. This diminishes the influence of weakly impacting parameters. In L1_REFIT, the L1 penalty was imposed exclusively on parameters prefixed with a_, b_, h_, or delta_ through qL1reg. After L1 fitting, the regularized parameters with absolute values less than 1e-6 were discarded (assigned p = 0, excluded from the fit by qFit = 0, and limited by lb = ub = 0). The model was then rebuilt from the adapted interaction matrix, where the former non-zero values are replaced with zero without L1 to derive the final Chi-2 value and the model selection criteria (AIC, AICc, and BIC), with degrees of freedom calculated as ndata − k, where k is the number of fitted parameters, and ndata is the number of data points.

## Additional Methods

The following two methods are extensions of model analysis but were not used in the manuscript. Subgraph analysis examines the structure among all poorly matched genes, while upstream analysis specifically identifies the direct and indirect regulators of a single target gene.

### Upstream root-cause analysis and two-hop expansion

Based on this analysis, we determine specifically why certain genes are poorly fitted to the model. To do this, we identify the most important direct and indirect regulators of a poorly adapted target gene and examine whether these regulators themselves are well or poorly adapted and how active they are at the respective time point. This results in a list of candidates, ranked by relevance, that are most likely to be the cause of the error and thus serve as a starting point for adjustments to the network structure or the model assumptions. Unlike the approach outlined in the main manuscript, which uses the Chi-2 value to determine which genes (nodes) need to be modified and how they should be altered, this approach offers a different perspective. In this supplementary approach, the focus is on the regulation of the upstream genes rather than on the gene with the highest Chi-2 value.

To investigate potential solutions to high misfit, we provide a targeted root-cause analysis framework (‘upstream_root_cause.py’) that integrates the following: (i) the directed network adjacency matrix, (ii) per-gene Chi-2 contributions from the model fit, and (iii) the required regulatory change (i.e., more activation or inhibition) for a selected target gene (script: upstream_analysis.py). We hypothesize that correcting upstream inconsistencies could resolve downstream misfits.

We interpreted the adjacency matrix (‘extended_adjacency_matrix.csv’) in the regulator → target orientation. Positive values indicate activation, negative values indicate inhibition, and zero indicates no interaction. Although the matrix may contain values such as 0, ±1, and ±2, edge magnitudes were not interpreted as interaction strengths. Instead, edges were treated as binary existence with a sign indicating activation or inhibition. Gene identifiers were normalized by removing suffixes, such as ‘_obs’, and by truncating spaces, to ensure consistent mapping between files.

Per-gene Chi-2 contributions were imported from 'chi2_sums_per_variable_full.csv'. The target gene and the required regulatory direction (activation or inhibition) were read from the first row of the 'gene_with_regulation.csv' file (the gene name is in the first column, and the required direction is in the last column).

Direct upstream regulators of the target were extracted as non-zero incoming edges from the target column in the adjacency matrix and were then separated into activators (w > 0) and inhibitors (w < 0). The regulators were then prioritized using a composite score:

Score(R→T) = I(w(R,T)≠0) × χ²(R) × state(R),

where I(·) indicates edge existence, χ²(R) represents the regulator's Chi-2 contribution, and state(R) is an optional, based activity term in [0, 1]. The state was derived from the long-format file ‘chi2_full.csv’ by selecting the time point at which the target exhibits maximum Chi-2 and using either ExpData or SimulatedData as the state source. If no state was available, state(R) was set to 1.

To quantify whether the target is structurally dominated by activating or inhibiting inputs, we computed a regulatory balance based on the number of incoming edges. +count = #(w > 0) and −count = #(w < 0), with net balance (+count − −count). This highlights cases with missing inhibitory control ('missing brakes') or an imbalance towards activating inputs. If the required direction was absent from the network structure (e.g. inhibition was required, but there were no inhibitory edges), this was reported as a structural inconsistency. Regulators of the opposite direction were then inspected as potential drivers of the discrepancy.

Two-hop extensions were performed to capture indirect upstream drivers by analyzing the upstream regulators (U) of selected high-priority one-hop regulators (R), yielding paths U → R → T. Two-hop candidates were scored using the same scheme: I(w≠0) × χ²(U) × state(U). Seed nodes for the two-hop expansion were selected based on the required direction, if available. If the required direction was present, but the corresponding regulators were inactive due to having a very low state, the seeds were switched to the opposite direction (e.g., inhibitors) to identify potential blocking mechanisms.

In summary, the script is designed to quickly identify which upstream genes in the network are most likely to be the cause of a high model error (high Chi-2) in a gene. It takes the target gene and searches the adjacency matrix for direct regulators (activators and inhibitors). It then evaluates these regulators based on whether they are a poor fit (i.e., have a high Chi-2 value) and are active at the relevant time point (as specified by ExpData/SimulatedData). If the target gene needs “more activation” but the activators are inactive, the script automatically switches to examining the inhibitors as possible blockers. A 2-hop step is then performed to identify which genes drive or inhibit these most important regulators. The result is a priority list that clearly shows who drives whom and who blocks whom. This allows you to target the most likely causes by checking the network structure, adding edges, or adjusting model assumptions/parameters, rather than trying things out unsystematically in the network.

### Subgraph analysis of non-fitting genes

We use this subgraph analysis to examine poorly fitting genes not in isolation, but within the context of their network. In doing so, we check whether they occur in isolation, in simple chains, in cycles, or are connected within more complex substructures. This makes it easier to decide where to start improving the model and avoids correcting only downstream effects when the actual cause lies further upstream in the same subnetwork.

Since nodes are coupled, the change in one node can influence the expression values of other nodes. For example, in a chain of regulating genes that are not all well-regulated, regulating the most upstream one might already improve the downstream ones. Moreover, if we first correct the downstream ones, their values can worsen once we correct the upstream ones. For this purpose, we provide a framework that investigates substructures among all badly fitting nodes, which is helpful to see where to start the improvement. For this purpose, we use the adjacency matrix G and a list of nodes L that contains all genes that have a Chi-2 value beyond a certain threshold, e.g., the mean Chi-2 value per gene for a model that is not rejected. Subgraphs are formed according to the node list L in every possible variation. The Python packages itertools and defaultdict (from collections) were used (‘subgraph.py’).

The function ‘build_induced_subgraph’ (G, L) generates subgraphs for the nodes in L using the adjacency matrix. To accomplish this, a directed adjacency list (subG) and an additional undirected variant (undirected_subG) are created. The latter is used to identify related components independently of arrow direction. The ‘find_undirected_components’ function (undirected_subG, L) uses a depth-first search (DFS) algorithm to find disjoint subgraphs.

For each identified component, the function evaluates whether it is a single node, a simple directed path (correction should start at the most upstream one), a simple directed cycle (correction can start everywhere in the cycle), or a more complex structure where an individual correction strategy needs to be worked out. Three functions are used to investigate the substructures for this purpose. The function ‘is_directed_simple_path’ (subG, comp) determines if the nodes form a path with in- and out-degrees corresponding to the start, end, and intermediate nodes. The function ‘is_single_directed_cycle’ (subG, comp) determines if each node has exactly one incoming and one outgoing edge, forming a single closed cycle. The function ‘contains_multi_node_cycle’ (subG, nodes) ensures that there are no directed cycles involving two or more nodes when a path is expected (self-loops are ignored in the implementation). When a path or cycle is detected, the functions ‘order_directed_path’ (subG, comp) and ‘order_single_cycle’ (subG, comp) ensure that the nodes are in the correct order.

The actual categorization into one of the four possible categories is performed using the ‘classify_component’ function (subG, comp) (1 = single node, 2 = simple path, 3 = cycle, and 4 = everything else). These steps are implemented for the entire collection of nodes using the ‘process_graph’ (G, L) function.

Finally, the ‘translate_results’ function (classification, index_to_gene) puts everything together. It converts node indices (integers representing positions in the adjacency matrix, each connected component ‘comp’ is a set of such indices) into gene names using a dictionary mapping (index_to_gene). This allows the results to be presented in a readable form (‘subgraph.csv’, <https://github.com/Sunbio1/DataXflowGen>).

Finally, please note that the 2-hop script carries out a root cause analysis for a single target gene. It ranks direct regulators (1-hop) and extends the top candidates to include their upstream regulators (2-hop), assigning a score based on edge existence × χ². In contrast, the subgraph script does not evaluate causes or scores, but iterates over all node subsets and classifies their components according to structure only (isolated, path, cycle, or other), writing this information to a ‘subgraph.csv ’ file.

# List of Figures

[Figure S 1: Second step of iterative GenAI modeling. 2](#_Toc222335403)

[Figure S 2: Third step of iterative GenAI modeling. 3](#_Toc222335404)

[Figure S 3: Fourth step of iterative GenAI modeling. 4](#_Toc222335405)

[Figure S 4: Fifth step of iterative GenAI modeling. 5](#_Toc222335406)

[Figure S 5: Sixth step of iterative GenAI modeling. 6](#_Toc222335407)

[Figure S 6: Prompt-based approach to generating a fibroblast-specific senescence gene interaction network. 8](#_Toc222335408)

[Figure S 7: One-shot model prompt for automated use of the analysis pipeline with genes of interest. 9](#_Toc222335409)

[Figure S 8: Prompt for automated annotation of gene interactions in fibroblast senescence. 9](#_Toc222335410)

[Figure S 9: Back loop prompt for automated identification of upstream regulators for target genes. 10](#_Toc222335411)

[Figure S 10: Simple back loop prompt for identifying causal upstream regulators in senescence. 11](#_Toc222335412)

# List of Tables

[Table SR 1: Fitting results of iterative modeling via GenAI response. 7](#_Toc222335805)
